# Chrysin as a Bioactive Scaffold: Advances in Synthesis and Pharmacological Evaluation

**DOI:** 10.3390/ijms26199467

**Published:** 2025-09-27

**Authors:** Chae Yun Jeong, Chae-Eun Kim, Eui-Baek Byun, Jongho Jeon

**Affiliations:** 1 School of Chemical Engineering and Applied Chemistry, Kyungpook National University, Daegu 41566, Republic of Korea; connie3114@knu.ac.kr (C.Y.J.); ce0916@knu.ac.kr (C.-E.K.); 2 Advanced Radiation Technology Institute, Korea Atomic Energy Research Institute, Jeongeup 56212, Republic of Korea; ebbyun80@kaeri.re.kr

**Keywords:** chrysin, drug design, synthetic strategies, pharmacological activities

## Abstract

Chrysin (5,7-dihydroxyflavone) is a flavonoid widely distributed in propolis, honey, and various plant sources. It exhibits a wide range of pharmacological activities, including anti-inflammatory, antioxidant, anticancer, antimicrobial, and anti-diabetic effects. However, its clinical translation is hampered by poor aqueous solubility, low bioavailability, and rapid metabolic clearance. To address these limitations and expand the chemical space of this natural scaffold, extensive synthetic efforts have focused on generating structurally diverse chrysin derivatives that possess improved drug-like properties. This review systematically categorizes synthetic methodologies—such as etherification, esterification, transition-metal-mediated couplings, sigmatropic rearrangements, and electrophilic substitutions—and integrates them with corresponding biological outcomes. Particular emphasis is placed on recent (2020–present) advances that directly link structural modifications with pharmacological enhancements, thereby offering comparative structure–activity relationship (SAR) insights. In addition, transition-metal-catalyzed C–C bond-forming reactions are highlighted in a dedicated section, underscoring their growing role in accessing bioactive chrysin analogs previously unattainable by conventional chemistry. Unlike prior reviews that mainly summarized biological activities or broadly covered flavonoid scaffolds, this article bridges synthetic diversification with pharmacological evaluation. It provides both critical synthesis and mechanistic interpretation. Overall, this work consolidates current knowledge and suggests future directions that integrate synthetic innovation with pharmacological validation and address pharmacokinetic challenges in chrysin derivatives.

## 1. Introduction

**Figure 1:**
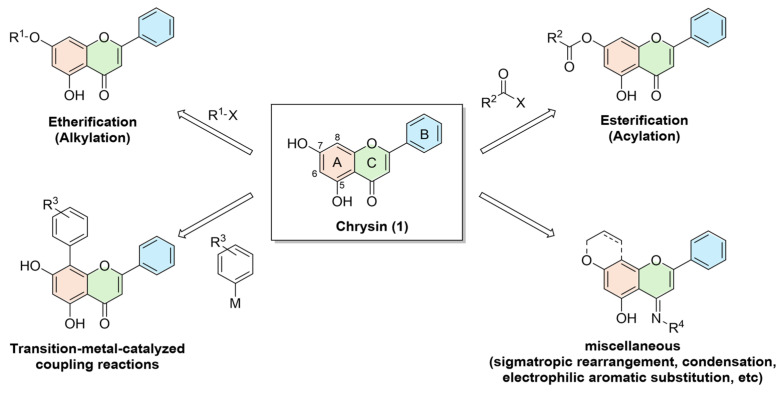
Representative strategies for the synthesis of chrysin derivatives.

Flavonoids are a structurally diverse class of polyphenolic compounds that have been extensively investigated for pharmacological applications. Representative examples include quercetin, one of the most abundant dietary flavonoids with strong antioxidant, anticancer, antiviral, and anti-inflammatory activities. Another is rhoifolin, which has recently garnered interest due to its potent cytotoxic and anticancer effects mediated by pro-apoptotic and anti-metastatic mechanisms [1,2]. More recently, emerging flavonoids such as prunin have also been highlighted for their anticancer potential, as summarized in recent reviews [3]. Within this broad chemical family, chrysin (5,7-dihydroxyflavone) has garnered particular attention due to its distinctive structural and pharmacological advantages. It has been identified in propolis, honey, and various plant extracts and has demonstrated a wide range of biological activities in both in vitro and in vivo studies [4,5,6,7]. Structurally, chrysin comprises two phenyl rings (A and B) and a heterocyclic ring (C) fused to the A ring, with hydroxyl groups located exclusively at the 5 and 7 positions of the A ring (Figure 1). This unique configuration imparts characteristic physicochemical properties that underlie its diverse biological activities. Chrysin has attracted considerable attention over the past few decades due to its broad spectrum of pharmacological effects. These include anti-inflammatory, antioxidant, anticancer, anxiolytic, and antimicrobial activities [5,8,9,10,11]. These bioactivities have been extensively validated through both in vitro and in vivo models. Owing to its multi-faceted pharmacological potential, chrysin has emerged as a structurally attractive and biologically relevant scaffold for the rational design of therapeutic agents and nutraceutical formulations [12,13].

Despite these promising attributes, the clinical translation of chrysin remains limited. Its poor aqueous solubility (0.058 ± 0.04 mg/mL at pH 7.4), low intestinal absorption, rapid metabolic degradation, and prompt systemic elimination collectively result in extremely low oral bioavailability [14,15,16]. These limitations are predominantly attributed to chrysin’s pronounced vulnerability to phase II metabolic enzymes, most notably UDP-glucuronosyltransferases (UGTs), which mediate extensive glucuronidation and sulfonation reactions that accelerate its clearance from the body [17,18]. To address these challenges, extensive efforts have been devoted to the structural modification of chrysin through various organic synthetic strategies. These modifications have generally pursued three major objectives: (i) to overcome intrinsic pharmacokinetic limitations of chrysin, including low bioavailability; (ii) to optimize its inherent pharmacological properties, thereby enhancing potency, selectivity, and overall therapeutic efficacy; and (iii) to broaden its therapeutic potential by introducing new biological activities not present in the native flavone.

This review aims to provide a comprehensive and critical overview of recent advances in the synthesis of chrysin derivatives and their biological evaluation. Specifically, we introduce representative synthetic approaches used to generate structurally modified chrysin analogs. We also highlight the pharmacological activities reported for these compounds across various biological systems. The majority of studies discussed herein were published within the past five years; however, earlier foundational works are also introduced where appropriate to contextualize key developments. Unlike previous reviews that mainly focused on either pharmacological properties or broad classes of flavonoids [4,5,6,7], this article emphasizes recent (2020–present) progress by systematically categorizing synthetic methodologies of chrysin derivatives into distinct reaction classes, ranging from classical O-functionalization to modern transition-metal-catalyzed couplings and even radiation-driven transformations. Particular attention is given to how specific structural modifications alter biological activities, thereby offering comparative SAR insights across therapeutic domains. Furthermore, we dedicate a separate section to transition-metal-catalyzed coupling reactions. This allows us to highlight how state-of-the-art synthetic chemistry has enabled access to novel chrysin scaffolds with promising pharmacological potential. Such scaffolds were previously inaccessible through conventional transformations. By bridging advances in synthetic methodology with critical pharmacological evaluation, this review seeks to provide meaningful insights into the therapeutic potential of chrysin-based derivatives and their future prospects in drug development.

## 2. Synthetic Approaches Toward Structurally Diverse Chrysin Derivatives

To provide a comprehensive overview of chrysin-based chemical modifications, we categorized the synthetic methodologies for generating structurally diverse derivatives according to the type of chemical transformation employed. These classifications are shown in Figure 1. These strategies encompass both classical and contemporary approaches, enabling targeted functionalization of the chrysin scaffold to enhance its pharmacological properties and expand its therapeutic potential. There have also been reports of designing and synthesizing structurally diversified chrysin derivatives through the sequential application of these reaction pathways.

### 2.1. Etherification (Alkylation)

Etherification methodologies involve the formation of ether linkages via nucleophilic substitution or direct alkylation reactions using suitable electrophilic reagents such as alkyl halides or alkyl tosylates under basic conditions [19]. These reactions primarily target the phenolic hydroxyl groups at the 5- and 7-positions of chrysin [20]. Due to intramolecular hydrogen bonding between the 5-OH group and the adjacent carbonyl moiety, the 7-OH group is generally more accessible and reactive. As a result, it is the preferred site for electrophilic alkylation [21]. Under conditions employing excess electrophile (typically more than two equivalents), both hydroxyl groups can be alkylated, allowing for further structural diversification. The intermediates synthesized through this reaction are often utilized as key building blocks for the construction of structurally complex final products [22].

### 2.2. Esterification (Acylation)

Esterification reactions of chrysin typically involve acylation of the phenolic hydroxyl groups using carboxylic acids or their activated derivatives, such as acid chlorides [23]. Coupling agents such as ethyl(dimethylaminopropyl)carbodiimide (EDC) are commonly employed to facilitate ester bond formation under mild conditions. Alternatively, acid chlorides can react directly with hydroxyl groups in the presence of suitable bases, enabling efficient synthesis of ester derivatives [24]. Both etherification and esterification have been widely adopted as foundational strategies for the preparation of chrysin analogs due to their simplicity and versatility [25].

### 2.3. Transition-Metal-Mediated Coupling Reactions

Transition-metal-catalyzed coupling reactions represent a powerful tool for constructing novel carbon–carbon bonds within polyaromatic frameworks such as flavonoids [26,27,28]. These methodologies typically begin with the functionalization of flavonoids to introduce reactive intermediates bearing carbon–halogen (C–I, C–Br) or carbon–boron (C–B) bonds. Subsequent cross-coupling with aryl substrates is mediated by transition metal catalysts, predominantly palladium-based systems [29,30]. These reactions offer high regioselectivity, modularity, and synthetic efficiency, and have been extensively applied in the diversification of flavonoid scaffolds. Detailed synthetic strategies and biological evaluations of chrysin derivatives prepared via transition metal catalysis will be discussed in a separate section (Section 4).

### 2.4. Miscellaneous Transformations

This category encompasses a range of alternative synthetic routes that enable substantial structural modifications of chrysin beyond conventional alkylation, acylation, or metal-catalyzed coupling strategies. Representative examples include sigmatropic rearrangements (e.g., Claisen rearrangement) [31], electrophilic aromatic substitution reactions [32,33], and condensation methodologies. Sigmatropic rearrangements offer a regioselective approach for introducing prenyl or allyl groups into the aromatic core [31,34]. Electrophilic aromatic substitution provides a method for the synthesis of halogenated, nitrated, or sulfonated derivatives, which frequently exhibit altered electronic distribution on the aromatic backbone. Condensation reactions such as hydrazone or Schiff base formation at the C-4 carbonyl provide rapid access to extended π-conjugated systems. These reactions are typically conducted under mild, catalyst-free conditions and allow facile incorporation of pharmacophoric fragments. Finally, metal chelation and complexation exploit the hydroxyl and carbonyl groups of chrysin as donor sites to coordinate with transition metals such as Cu, Cr, Fe, or Ru. These coordination reactions not only diversify the chemical architecture but also yield organometallic scaffolds with distinct redox and electronic properties that are not accessible through purely organic transformations [35,36].

The structural diversification strategies described above have generated a broad spectrum of chrysin derivatives, which in turn provide the foundation for evaluating their pharmacological activities.

## 3. Chrysin Derivatives and Their Biological Activities

Chrysin derivatives obtained through chemical modification have been reported to possess diverse pharmacological properties, including anti-tumor, anti-inflammatory, antioxidant, antimicrobial, and anti-diabetic activities.

### 3.1. Antitumor Activity

**Scheme 1:**
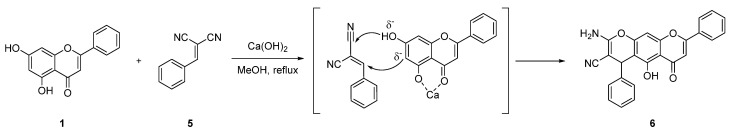
Synthesis of chrysin derivative **6**.

The Phaosiri group reported the chemical modification of chrysin at the 7-hydroxyl position through the introduction of various substituents, including alkyl, benzyl, carbonyl, and amino groups, leading to a focused library of synthetic derivatives [37]. Among these, compounds **2a** and **2b** (ethyl and propyl derivatives, respectively) exhibited strong pan-histone deacetylase (HDAC) inhibition (92–93% at 100 µM), while compound **2c**, bearing an α-substituted carboxylic acid side chain, achieved nearly complete inhibition (99% at 100 µM) with selective HDAC8 binding in molecular docking studies. Importantly, **2c** also demonstrated notable antiproliferative activity against HeLa cells (IC_50_ = 13.91 µM) and increased histone H3 acetylation, indicating epigenetic modulation as a potential anticancer mechanism. Small alkyl substituents at the 7-position provided broad HDAC inhibition but limited translation into cellular potency, whereas the α-substituted carboxylate side chain markedly enhanced both enzyme selectivity (HDAC8) and antiproliferative activity. This suggests that polar, bulky substituents at 7-OH can improve target specificity and epigenetic modulation compared with simple alkylation. Chen et al. reported the synthesis of ten chrysin derivatives through acylation of the 7-hydroxyl group with various aromatic and long-chain acyl chlorides under mild conditions using triethylamine as a base [23]. Compound **3**, a long-chain myristoyl chrysin derivative with a more flexible structure, exhibited markedly improved solubility and the most potent cytotoxicity against HepG2 liver cancer cells, with an IC_50_ of 14.79 μM—approximately 5-fold more active than chrysin itself (IC_50_ = 74.97 μM). The introduction of long flexible acyl chains increased lipophilicity and solubility, thereby improving cellular uptake and potency. This indicates that hydrophobic ester substituents at 7-OH can overcome the poor physicochemical profile of native chrysin and translate into stronger cytotoxic effects. Mayer et al. introduced an N-phenylchloroacetamide moiety at the 7-position of chrysin and subsequently employed a Smiles rearrangement to obtain novel 7-aminochrysin derivatives with a diphenylamine scaffold [22]. Among these, compound **4** exhibited markedly enhanced antitumor activity at the nanomolar level, with 50% growth inhibition concentration (GI_50_) values of 0.03 µM against breast cancer cells (MCF-7) and 0.060 µM against colon cancer cells (HCT-15), demonstrating superior potency compared with the parent chrysin. The rearrangement to a diphenylamine scaffold introduced extensive aromatic conjugation and stronger π–π interactions, resulting in nanomolar potency. This reveals that bulky aromatic extension at the 7-position dramatically enhances cytotoxic activity beyond what is achievable with simple alkyl or acyl groups. The Zhou group reported the regioselective synthesis and biological evaluation of novel 2-amino 3-cyano chromene–chrysin hybrids as anticancer agents [38]. These compounds were efficiently prepared through a Ca(OH)_2_-mediated cascade Michael/cyclization reaction between chrysin and arylidenemalononitriles (Scheme 1).

**Scheme 2:**
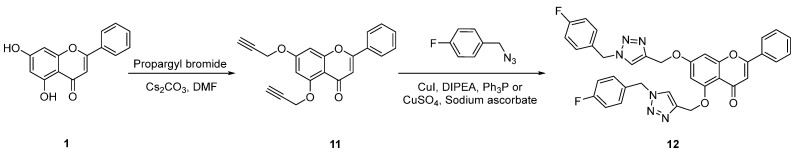
Synthesis of chrysin derivative **12** containing two 1,2,3-triazole units.

Among the derivatives synthesized, compound **6** exhibited the highest anticancer activity, showing potent cytotoxicity against K562 leukemia cells (IC_50_ = 6.41 µM) by inducing apoptosis via intrinsic mitochondrial pathways involving the regulation of Bax and Bcl-2 proteins. The incorporation of a chromene moiety with cyano and amino functionalities reinforces electron-withdrawing and hydrogen-bonding interactions, thereby favoring mitochondrial apoptosis. This indicates that fused heteroaryl substituents can broaden the pharmacophoric landscape of chrysin. Al-Oudat et al. reported the synthesis and biological evaluation of novel chrysin–de-allyl PAC-1 hybrid analogs as anticancer agents [39]. These compounds were synthesized via a three-step process involving alkylation, hydrazide formation, and hydrazone coupling reactions. Among the synthesized analogs, compounds **7a** and **7b** exhibited the most potent antiproliferative activities against triple-negative breast cancer (MDA-MB-231) cells, with IC_50_ values of 5.98 µM and 9.40 µM, respectively. Mechanistic studies demonstrated that both compounds induced apoptosis, caused cell cycle arrest at the G2 phase, and activated the intrinsic mitochondrial apoptotic pathway involving Bak upregulation, cytochrome c release, and caspase activation. The presence of hydrazone linkers connected to the PAC-1 pharmacophore proved crucial for activity, while differences in phenolic substitution patterns modulated potency. These findings emphasize that hybridization with apoptosis-inducing motifs enhances chrysin’s anticancer efficacy. A novel series of porphyrin–chrysin derivatives were developed as photosensitive anticancer agents, synthesized through sequential porphyrin preparation, substitution, and chlorination reactions [40]. Among these, compound **8** exhibited the highest photodynamic activity, with IC_50_ values of 6.26 μM (HeLa) and 23.37 μM (A549). Biological evaluations indicated that free-base porphyrin derivatives bind to ct-DNA through surface self-stacking, whereas zinc metalloporphyrins bind via intercalation, with binding strength positively correlating with anticancer efficacy. Structural analyses further indicated that enhancing positive charge, introducing electron-withdrawing groups, and modulating steric bulk improve their anticancer activities. The data demonstrate that electronic, steric, and chain-length tuning of porphyrin substituents directly influence the DNA-binding mode and phototoxic efficiency. Positively charged and electron-withdrawing groups, together with optimal chain length, markedly enhance ROS generation and cytotoxicity. Porphyrin–chrysin derivatives were synthesized and evaluated for photosensitizing and antitumor activities against human cancer cell lines [41]. These conjugates exhibited strong absorption in the Soret and Q bands and generated singlet oxygen upon light activation. Biological evaluation against MGC-803 (gastric) and HeLa (cervical) cancer cell lines revealed significantly enhanced cytotoxicity under light conditions. Notably, compound **9** showed the most potent photodynamic antitumor activity (IC_50_ = 26.51 μM in HeLa), surpassing chrysin and the reference drug 5-fluorouracil. Variation in porphyrin metallation revealed that free-base derivatives outperform metalated analogs in photodynamic efficacy. This highlights that subtle structural changes in the chromophore core strongly dictate singlet oxygen generation and cytotoxic performance. Novel flavonoid nitrogen mustard derivatives, derived from genistein and chrysin, were synthesized and evaluated for antitumor activity against several human cancer cell lines, including HeLa, A549, HepG2, MCF-7, SH-SY5Y, PC-3, and DU145 [42]. Among these derivatives, compound **10** exhibited remarkable antiproliferative activity, notably surpassing the efficacy of melphalan, with IC_50_ values as low as 1.43 µM against HeLa cells. Mechanistic studies indicated that compound **10** could effectively induce apoptosis and arrest the cell cycle at the G2/M phase in HeLa cells, possibly via disruption of mitochondrial membrane potential. These findings clearly demonstrate that linker length is a crucial determinant of potency, with an optimal three-carbon bridge conferring maximal cytotoxicity. Thus, rational tuning of spacer units is an effective SAR lever in mustard–flavonoid hybrids. The Keglevich group developed a series of flavone–1,2,3-triazole hybrids by coupling chrysin and kaempferol with substituted 1,2,3-triazole moieties via Cu(I)-catalyzed azide-alkyne cycloaddition (Copper-catalyzed click reaction) [43]. These hybrids were synthesized through propargylation followed by Cu catalyst-mediated triazole formation (Scheme 2).

**Scheme 3:**
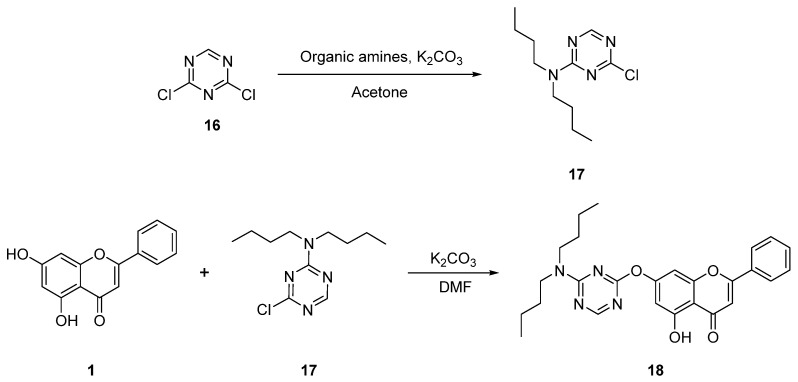
Synthesis of chrysin derivative **18**.

The compounds exhibited promising antiproliferative activities against various human cancer cell lines in the NCI-60 panel. In particular, the bis-substituted chrysin derivatives demonstrated significant anticancer potency, surpassing the efficacy of reference drugs in some cases. Compound **12** showed notable potency with a sub-micromolar IC_50_ value (0.733 µM) against HeLa cells, suggesting its potential for further optimization as a novel anticancer lead. The comparative data reveal that fluorine substitution strengthens electron-withdrawing interactions and stabilizes binding, whereas nitro groups negatively affect cytotoxic potency. Multiple triazole linkages promote hydrogen bonding and π-stacking, producing consistently higher activity than single-linker derivatives. Copper-catalyzed click reaction was utilized in a study reported by Noole et al., in which a series of novel 1,2,3-triazole derivatives of chrysin were synthesized by linking various aryl and alkyl azides to the 5-position of chrysin through a propargyl ether moiety [44]. The resulting compounds were evaluated for their in vitro anticancer activity against several cell lines. Among them, compound **13** (bearing a phenyl-substituted triazole) exhibited the most potent activity, with IC_50_ values of 10.8 μM in PC-3 cells and 20.5 μM in MCF-7 cells, outperforming the reference drug doxorubicin in MCF-7 cells. This indicates that aryl substituents on triazole units are more favorable than alkyl analogs for cytotoxic potency, likely due to enhanced π–π stacking and hydrophobic interactions. The click chemistry strategy was also employed in the study reported by Tial et al. [45]. The anticancer activities of the synthesized compounds were evaluated against six cancer cell lines, including MGC-803, BEL-7402, HepG2, HeLa, A549, and SGC-7901. Notably, compounds **14a** (bearing a 3-fluorophenyl substituent) and **14b** (bearing a 2-chlorophenyl substituent) exhibited superior cytotoxicity against the gastric cancer cell line MGC-803, with IC_50_ values of 18.40 and 5.92 μM, respectively, which is better than both the parent compound chrysin and the positive control 5-fluorouracil. The results indicate that halogen substituents on the aryl-triazole ring improve activity, with chlorine at the ortho-position being particularly effective. This highlights the importance of electronic effects and positional isomerism in enhancing the potency of triazole-bearing derivatives. Conjugates comprising vindoline and chrysin were reported by the Mayer group, synthesized by coupling vindoline with chrysin via either an amide or ester linkage [46]. The desired hybrid was successfully synthesized by first converting chrysin into a carboxylic acid derivative, then coupling it with 10-aminovindoline. Additionally, another hybrid was prepared by linking chrysin to vindoline at position 17 via a flexible linker. Among the synthesized compounds, hybrid **15** exhibited the most potent anticancer activity, with low micromolar GI_50_ values against several human cancer cell lines in the NCI60 panel. The hybridization with vindoline markedly boosted potency, suggesting pharmacophore synergy. Flexible linkers appeared to facilitate optimal spatial orientation, improving activity over direct rigid coupling. Xue et al. reported a series of chrysin-1,3,5-triazine derivatives, synthesized via nucleophilic substitution reactions between chrysin and substituted 1,3,5-triazines [47]. 2,4-Dichloro-1,3,5-triazine was first reacted with various amines, followed by coupling of the resulting intermediates with chrysin under basic conditions to yield the target hybrids (Scheme 3).

Among these compounds, derivative **18** exhibited the most potent anticancer activity, particularly against HeLa cervical cancer cells (IC_50_ = 9.86 ± 0.37 μM), surpassing the activities of both cisplatin (IC_50_ = 28.09 ± 0.47 μM) and chrysin (IC_50_ = 29.51 ± 0.51 μM). Compound **17** effectively inhibited the proliferation, invasion, and adhesion of HeLa cells and demonstrated significant tumor growth inhibition in vivo. Substitution with dichloro-1,3,5-triazine rings introduced strong electron-withdrawing effects, enhancing binding affinity and cytotoxicity. Comparative results indicate that increased electronic deficiency at the heteroaryl moiety correlates with superior antiproliferative performance. Similarly, Yu’s group reported the synthesis of a series of chrysin derivatives through nucleophilic substitution reactions between chrysin and dichloropyrimidines [48]. A total of 17 compounds were prepared and evaluated for their antiproliferative activity. Among them, compound **19a** exhibited notable cytotoxicity across four cancer cell lines (A549, HepG2, MCF-7, and PC-3), while **19b** was the most potent against HCT-116 cells (IC_50_ = 4.83 µM). Activity differences among the pyrimidine hybrids suggest that specific halogen substitutions and heteroaryl electronic effects govern potency. Compound **19b**’s superior activity against HCT-116 highlights the value of precise substituent positioning on the pyrimidine scaffold. In 2020, a series of chrysin amino acid derivatives were synthesized by modifying the 7-hydroxyl group of chrysin with heptanoyl-linked L-amino acids with the aim of enhancing anticancer potency and tumor selectivity [49]. Among the synthesized compounds, **20** (chrysin conjugated with L-isoleucine) showed the most potent antiproliferative activity, especially against the gastric cancer cell line MGC-803, with an IC_50_ of 24.5 μM—superior to both chrysin and 5-fluorouracil. Conjugation with hydrophobic amino acids improved cell permeability and selectivity. The results emphasize that combining lipophilic linkers with polar amino acid residues can balance solubility and activity to yield enhanced anticancer efficacy. Later, Li et al. synthesized a series of 16 chrysin-based amino acid derivatives by introducing various L-amino acid fragments at the 7-position of chrysin through an alkylation–amidation strategy [50]. The key intermediate, obtained by O-alkylation of chrysin with ethyl 2-bromooctanoate, was hydrolyzed to the corresponding acid and subsequently coupled with different amino acid methyl ester hydrochlorides using EDCI/HOBt coupling. Among the synthesized compounds, **21** (chrysin conjugated with L-leucine methyl ester) exhibited the most potent antiproliferative activity, particularly against MDA-MB-231 and MCF-7 breast cancer cells, showing superior IC_50_ values compared with 5-fluorouracil. Mechanistic studies demonstrated that **21** induced G2/M phase cell cycle arrest, triggered apoptosis via the mitochondrial pathway, inhibited Akt phosphorylation, and regulated the expression of Bcl-2 and Bax proteins. The study highlights that amino acid side chains contribute to both target interaction and solubility. In particular, leucine methyl ester conjugation enhanced mitochondrial apoptosis, suggesting that hydrophobic side chains improve potency by facilitating intracellular delivery and pathway modulation. Kamloon et al. designed and synthesized a series of 33 chrysin derivatives bearing various substituents—such as alkyl carboxylates, tertiary amines, and alkyl bromides—at the 7- or 5,7-hydroxy groups to enhance antiproliferative activity through putative histone deacetylase (HDAC) inhibition [51]. Guided by molecular docking simulations targeting the HDAC active site, they identified several compounds, notably **22a**, **22b**, and **22c**, that exhibited strong cytotoxicity against HeLa, HCT-116, A549, and MCF-7 cancer cell lines, with IC_50_ values in the low-to-mid micromolar range. The compound **21a** docking and Zn^2+^ chelation assays revealed favorable binding of these carboxylated derivatives to the catalytic pocket, including key interactions with Zn^2+^, supporting a potential HDAC-inhibitory mechanism. The results confirm that carboxylated and halogenated substituents enhance HDAC binding by coordinating with Zn^2+^. The comparative potency of **22a**–**c** highlights that chain length and polarity fine-tune cytotoxicity through modulation of active-site interactions. The Ning group reported the design and synthesis of novel chrysin derivatives bearing a 1*H*-benzimidazole-4-carboxamide pharmacophore at the C-7 position, aiming to enhance inhibitory activity against poly(ADP-ribose) polymerase 1 (PARP1) [52]. Among the synthesized compounds, **23** exhibited the most potent activity, with an IC_50_ of 108 nmol·L^−1^ for PARP1 and strong antiproliferative effects against BRCA-deficient breast cancer cell lines (HCC-1937, IC_50_ = 3.9 µM; MDA-MB-436, IC_50_ = 4.5 µM). In vivo, **23** significantly reduced tumor growth in xenograft models by downgrading PARP 1 expression. Introduction of a benzimidazole pharmacophore dramatically improved potency by strengthening hydrogen-bonding and π–π interactions with PARP1. This result underlines the utility of heteroaryl pharmacophore fusion for designing highly selective anticancer inhibitors.

Oggero et al. reported PEGylated derivatives of chrysin to improve its poor water solubility while preserving biological activity [53]. Using tosylated methoxy-polyethylene glycols (mPEG, 350–2000 g/mol), they selectively conjugated PEG chains at the C7-OH position. The 500 g/mol PEG conjugate **24b** exhibited the greatest solubility enhancement, formed stable nanoaggregates, and retained redox, antimicrobial, and cytotoxic activities. These findings demonstrate that PEG size is a critical determinant of drug-like optimization, with low-molecular-weight PEGylation offering a favorable balance of solubility and potency. Kulhari et al. developed PEGylated chrysin nanoparticles (PCNPs) **25** using PEG4000 with pH-sensitive linkers (succinoyl, cis-aconityl) [54]. These conjugates self-assembled into nanoparticles and released chrysin preferentially under acidic tumor microenvironments. Notably, the cis-aconityl system (PCNP-2) exhibited superior pH-triggered release and anticancer efficacy compared with both free chrysin and PCNP-1. These results demonstrated the importance of linker chemistry in controlling nanoparticle behavior and therapeutic performance.

In comparison to other delivery systems, PEG conjugation provides predictable improvements in aqueous solubility, stability, and circulation, with relatively simple chemistry. Micelles afford high encapsulation efficiency but often suffer from dilution-induced instability under physiological conditions [55]. Liposomes, validated in several clinical formulations, offer biocompatible encapsulation and targeted delivery [56]. Polymeric carriers such as PLGA nanoparticles enable sustained release and tunable pharmacokinetics, though concerns remain regarding degradation and clearance [57]. Collectively, PEGylation represents a straightforward and effective strategy, while micelles, liposomes, and polymeric nanoparticles serve as complementary but more complex alternatives for enhancing the therapeutic potential of chrysin derivatives. A comprehensive review of the recent advancements in flavonoid-based nanodelivery systems has recently been reported [58].

The Balaji group synthesized thirty spirooxindole carbamate derivatives of flavonoids (chrysin, oroxylin A, baicalein) via a one-pot three-component reaction using isatin, urea, and flavonoids, proceeding through Knoevenagel condensation and cyclization [59].

**Scheme 4:**
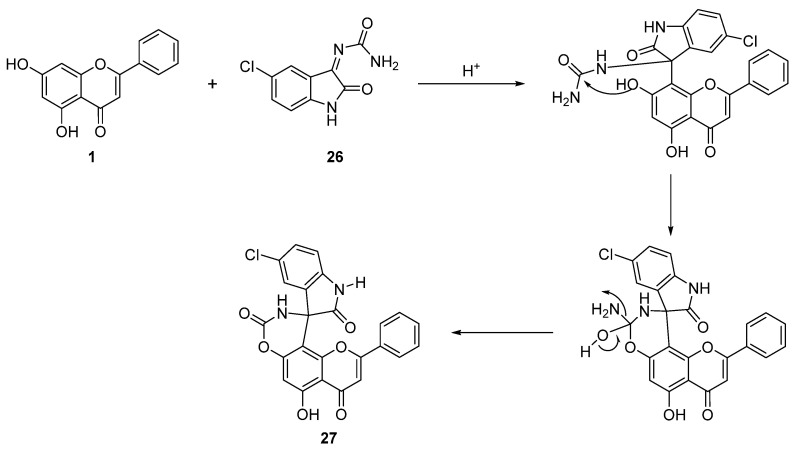
Plausible mechanism for the synthesis of chrysin derivative **27**.

Scheme 4 shows the plausible reaction mechanism for the formation of spirooxindole carbamate derivatives. Among them, chrysin-based compound **27** showed the most potent anticancer activity, with an IC_50_ of 2.50 ± 0.25 μM against HepG2 cells. It induced significant early apoptosis, reduced CD133^+^ cancer stem-like cells, and inhibited tumorsphere formation. Docking studies revealed strong interaction with CD133 (−10.2 kcal/mol), and in vivo xenograft studies confirmed its tumor growth suppression. The spirooxindole carbamate scaffold provided a rigid three-dimensional pharmacophore that enhanced CD133 binding and stemness inhibition. The carbamate group contributed to stability and apoptosis induction, demonstrating the advantage of heterocyclic fusion for targeting cancer stem-like cells. A chrysin thiazole derivative **28** was synthesized and identified as a potential toll-like receptor 4 (TLR4) ligand capable of reprogramming tumor-associated macrophages (TAMs) toward the anti-tumorigenic M1 phenotype [60]. Compound **28** activated the TLR4/NF-κB signaling pathway, promoted NF-κB/p65 nuclear translocation, and enhanced the release of pro-inflammatory cytokines. It also triggered type I interferon signaling, increasing IFN-α, IFN-β, and target genes NOS2, MCP-1, and IP-10 in macrophages. These effects were abolished in TLR4^−^/^−^ macrophages generated using CRISPR/Cas9, confirming TLR4 dependence. Molecular docking further supported **28** as a TLR4-binding ligand, suggesting its potential as a lead compound for cancer therapy. Incorporation of a thiazole moiety at the 7-position enabled direct TLR4 engagement, transforming chrysin into an immunomodulatory scaffold. The electron-rich heteroaryl group facilitated receptor binding, linking structural modification to macrophage reprogramming and antitumor immunity. The carbonyl group of chrysin is known to react with nitrogen-containing strong nucleophiles to form imine (C=N) linkages. A green synthetic approach was employed to develop chrysin hydrazone derivatives by condensing chrysin with various aryl hydrazines under solvent-free conditions using the ionic liquid [Et_3_NH][HSO_4_] as a catalyst [61]. Structural diversity was achieved by introducing different electron-donating and electron-withdrawing substituents on the phenylhydrazine moiety. Among them, **29a** (2-methoxyphenyl hydrazone) showed the most potent cytotoxicity against MCF-7 cells (IC_50_ = 101.44 µM), while **29b** (4-methoxyphenyl hydrazone) was most effective against HepG2 cells (IC_50_ = 166.19 µM), both outperforming chrysin itself. The position of methoxy substitution influenced cytotoxicity, suggesting that electron-donating groups at ortho or para positions can modestly enhance efficacy. Nevertheless, overall activity remained low, indicating that hydrazone linkages are less effective in generating potent anticancer derivatives.

The incorporation of chrysin into organometallic complexes is a promising strategy for enhancing its therapeutic potential. Metal coordination has been shown to enhance lipophilicity, redox properties, and electronic distribution [35,36]. This, in turn, has been demonstrated to increase membrane permeability, metabolic stability, and target affinity. These hybrids frequently exhibit multi-mechanistic anticancer activity with notably lower IC_50_ values and greater selectivity compared with free chrysin. Organometallic scaffolds have been shown to exert therapeutic effects through enhanced target binding and cross-linking, induction of reactive oxygen species, mitochondrial dysfunction, and cell cycle arrest. These effects have been widely observed in platinum, ruthenium, copper, and other transition-metal complexes [62,63]. The choice of metal center and ligands allows for precise modulation of potency, selectivity, and mechanism of action. This provides a versatile platform for the development of novel therapeutic agents. A representative example is provided by Break et al., who reported a novel chrysin–ferrocene Schiff base **30** synthesized by condensation with aminoferrocene [64]. The chrysin–ferrocene conjugate **30** displayed potent cytotoxicity against HepG2, HCT-116, and A549 cancer cells (IC_50_ ≈ 3 µM) with ~5-fold selectivity over normal MRC5 cells, inducing G1-phase cell-cycle arrest and caspase-dependent apoptosis. It also inhibited topoisomerase II comparably to etoposide, while in silico studies highlighted the critical role of the ferrocenyl moiety in drug-like interactions. The ferrocene unit acted as a redox-active pharmacophore, boosting ROS generation and Topo II inhibition. This illustrates how organometallic conjugation can synergize with the flavonoid scaffold to yield multi-targeted cytotoxicity with improved selectivity. In another study, Alem et al. synthesized and structurally characterized two novel heteroleptic Cr(III) complexes incorporating the natural flavonoid chrysin: **31**, a 1,10-phenanthroline–chrysin complex and **32**, a metformin–chrysin complex [65]. The complexes were prepared by reacting Cr(III) salts with chrysin and the respective co-ligands, and their structures were confirmed via spectroscopic analyses and single-crystal X-ray diffraction. Both complexes were evaluated for their anticancer properties and demonstrated higher cytotoxicity against human cancer cell lines (including MCF-7 and HeLa) compared with free chrysin, with **31** showing the strongest activity. Coordination with Cr(III) enhanced lipophilicity and electronic tuning, while the phenanthroline co-ligand provided superior binding and cytotoxicity compared with metformin. These findings confirm that ligand environment critically modulates metal–chrysin activity. Manzano et al. reported the synthesis of a series of half-sandwich Ru(II), Rh(III), and Ir(III) complexes incorporating O-alkylated chrysin ligands coordinated to η^5^-Cp metal fragments **33a-c** [66]. The resulting complexes exhibited potent cytotoxicity, particularly those containing Ir(III), which showed low-micromolar IC_50_ values against A2780, MCF-7, and HeLa cells, along with markedly higher selectivity over normal human dermal fibroblasts. Mechanistic studies indicated apoptosis via mitochondrial depolarization, ROS generation, and caspase activation. The comparative data emphasize that Ir(III) confers the greatest potency among the half-sandwich complexes, correlating with strong ROS induction and mitochondrial targeting. Additionally, variation in O-alkyl substituents modulated activity, showing that both the metal identity and organic ligand govern efficacy. Huang et al. developed a chrysin-derived difluoroboron complex **34** as the first example of a natural chrysin-based anion receptor [67]. In addition to its selective recognition of F^−^, H_2_PO_4_^−^, and AcO^−^ anions, Ch-FB displayed enhanced anticancer activity compared with the parent flavonoid. In vitro assays revealed that **34** inhibited the growth of MCF-7 and SKOV3 cells, with IC_50_ values of 29.7 μM and 31.9 μM, respectively, more than twofold improvement over chrysin. Coordination with difluoroboron altered electronic distribution and molecular planarity, strengthening interactions with cellular targets. This modification doubled cytotoxic potency, indicating that boron coordination chemistry can be exploited to improve the therapeutic profile of flavonoids.

### 3.2. Anti-Inflammatory Activity

**Scheme 5:**
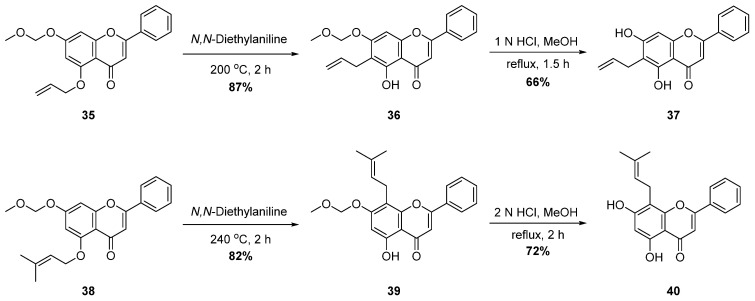
Claisen rearrangement of chrysin derivatives (**37**, **40**).

**Scheme 6:**
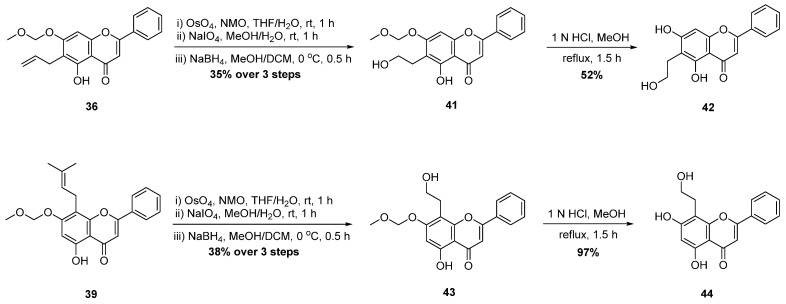
Synthesis of hydroxyethylated chrysin derivatives (**42**, **44**).

New chrysin derivatives were synthesized by the Jeon group using a Claisen rearrangement strategy to explore their anti-inflammatory potential [68]. Allyl or prenyl substituents introduced at the 5-hydroxyl group underwent thermally induced Claisen rearrangement, affording the C-6 isomer **36** from the allyl intermediate **35** and the C-8 isomer **39** via an initial C-6 shift from the prenyl analog **38** (Scheme 5). Further dihydroxylation, oxidative cleavage, and reduction converted **36** and **39** into primary alcohols **41** and **43**, followed by MOM deprotection to afford derivatives **37**, **40**, **42**, and **44** (Scheme 6). Among them, **40** and **42** showed the most potent Cyclooxygenase-2 (COX-2) inhibition (IC_50_ = 6.76 and 9.63 μM), surpassing chrysin (18.48 μM), and effectively reduced IL-6, TNF-α, and PGE_2_ levels in Lipopolysaccharide (LPS)-stimulated RAW264.7 cells. Molecular docking confirmed their strong COX-2 binding through hydrogen bonding and hydrophobic interactions, indicating that C-6/C-8 rearrangement followed by oxidation extends hydrophobic interactions and improves COX-2 selectivity and anti-inflammatory efficacy.

**Scheme 7:**
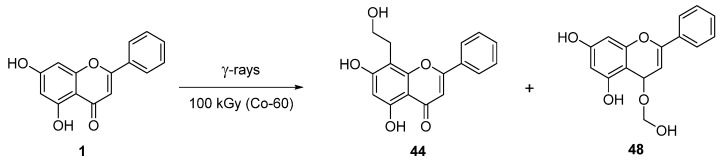
Synthesis of chrysin derivatives **44**, **48** through g-radiation of chrysin.

Lin et al. synthesized a series of sulfonylated chrysin derivatives by introducing various substituted aryls on the 7-(2-aminoethoxy) position of chrysin [69]. Among them, compound **45** exhibited the most potent activity, significantly inhibiting LPS-induced NO, TNF-α, IL-6, and IL-17A production in RAW264.7 macrophages and HaCaT keratinocytes, with an IC_50_ of 8.0 μM, outperforming chrysin (31.7 μM) and dexamethasone (19.5 μM). In an imiquimod-induced psoriasis model, **45** effectively reduced epidermal thickening and inflammatory cytokine expression via NF-κB and STAT3 modulation, underscoring its therapeutic potential in psoriasis-related skin inflammation. Collectively, bulky arylsulfonyl groups at C-7 improved potency, likely by introducing polar pharmacophores that favor target engagement and downstream NF-κB/STAT3 inhibition. In an effort to enhance the therapeutic potential of chrysin for inflammatory bowel disease (IBD), Zhao et al. designed and synthesized a series of chrysin derivatives incorporating α-lipoic acid (α-LA) via pharmacophore fusion using various linkers [70]. Among them, compound **46**—bearing a piperazine moiety—exhibited the most potent biological activity, significantly inhibiting TNF-α-induced monocyte adhesion to colon epithelial cells (IC_50_ = 4.71 μM), with superior potency compared with chrysin (>30 μM) and α-LA (12.7 μM). Mechanistic studies revealed that **46** suppressed ICAM-1 and MCP-1 expression, reduced ROS production, and inhibited NF-κB transcriptional activity in vitro. In a TNBS-induced colitis rat model, oral administration of **46** markedly attenuated disease symptoms and lowered pro-inflammatory cytokines (IL-6, IL-1β, TNF-α) by downregulating JAK2/STAT3 signaling. Fusion with α-lipoic acid, especially via a piperazine linker, significantly improved both anti-inflammatory potency and in vivo efficacy. This suggests that dual-pharmacophore hybrids can synergize to block multiple inflammatory pathways. Ávila-Román et al. synthesized two derivatives of chrysin-8-C-glucoside, **47a** and **47b**, to enhance stability and uptake [71]. Starting from chrysin-8-C-glucoside, extracted from M. rosei, they synthesized **47a** and **47b** through preacetylation and carbonylation, respectively. Both compounds significantly reduced ROS production and suppressed TNF-α, IL-1β, and COX-2 expression in LPS-stimulated THP-1 macrophages. Mechanistic studies revealed activation of the Keap1/Nrf2/HO-1 pathway, with compound **47a** showing the strongest activity, highlighting their dual anti-inflammatory and antioxidant potentials. Substitution at the C-8 glucoside with acetyl or carbonate groups enhanced stability and Nrf2 pathway activation. Acetylation, in particular, provided the best activity, linking improved metabolic stability to stronger dual antioxidant/anti-inflammatory effects. The Byun group successfully synthesized novel chrysin derivatives **44** and **48** through radiation chemistry by γ-irradiating a methanol solution of chrysin at 100 kGy using a cobalt-60 source (Scheme 7). As reported, one of the products, **48**, alleviated skin lesions and reduced inflammatory cytokines in a mouse model of atopic dermatitis, demonstrating in vivo anti-inflammatory efficacy [72]. Subsequent studies revealed that **44** exerted potent anti-inflammatory effects in LPS-stimulated RAW264.7 macrophages by upregulating Toll-interacting protein (Tollip), which suppressed NF-κB and MAPK activation and decreased TNF-α, IL-6, and NO production [73]. Moreover, **44** enhanced the expression of negative regulators such as TNFAIP3, contributing to the resolution of inflammation. Importantly, in vivo administration of **44** protected mice from endotoxin-induced lethal shock by reducing systemic cytokine levels and neutrophil infiltration, confirming its therapeutic potential against excessive inflammatory responses [74]. Radiation-driven structural rearrangements yielded unique derivatives with strong immunoregulatory capacity. Upregulation of Tollip and negative regulators illustrates that such non-classical modifications can produce compounds with distinct anti-inflammatory mechanisms compared with conventional substitutions.

### 3.3. Anti-Microbial Activity

Chrysin-based pyrimidine–piperazine hybrids have been developed as promising antimicrobial agents and systematically evaluated against a broad panel of pathogens [75]. Among several synthesized products, **49a** and **49b** exhibited the strongest antibacterial effects against *Escherichia coli* (*E. coli*) (MIC 6.25 and 12.5 µg/mL, respectively), which were better than standard antibiotics such as ampicillin, chloramphenicol, and ciprofloxacin. In addition, some derivatives demonstrated superior antifungal activity against *Candida albicans* (*C. albicans)* compared with griseofulvin. Structure–activity relationship analysis highlighted the importance of electron-withdrawing substituents and free phenolic hydroxyl groups for enhancing potency. Consistent with these findings, molecular docking revealed favorable binding of the active compounds to *E. coli* DNA gyrase (PDB ID: 1KZN) and *C. albicans* CYP51 (PDB ID: 5V5Z), with **49b** displaying the strongest binding affinities, respectively. Incorporation of piperazine with electron-withdrawing groups improved bacterial enzyme binding and potency. Retaining free phenolic hydroxyls was also essential, underscoring their role in stabilizing target interactions. Bhowmik et al. synthesized twenty-two novel chrysin derivatives through 7-OH functionalization, employing two synthetic strategies: a click reaction and condensation with various α-chloroacetamide derivatives [76]. These compounds were evaluated for antimicrobial and antibiofilm activity against *E. coli* MTCC 40. Among them, **50a**, **50b**, **50c**, and **51** exhibited the most potent effects, achieving over 92% biofilm inhibition at sub-MIC concentrations (93.57%, 92.14%, 92.14%, and 93.57%, respectively), exceeding that of chrysin (33.57%). In addition, these derivatives markedly suppressed *E. coli* motility and demonstrated improved dissolution profiles and higher bioavailability compared with chrysin. Introduction of triazole and α-chloroacetamide linkers at the 7-position strongly enhanced antibiofilm activity. Improved solubility and dissolution indicate that physicochemical optimization is tightly linked to antimicrobial efficacy. Xia et al. synthesized three xanthone and three flavone derivatives to evaluate their quorum-sensing inhibition in *Pseudomonas aeruginosa* (*P. aeruginosa)* [77]. The chrysin-derived phosphate ester **52** was the most active, with 100 μM significantly suppressing LasR-regulated virulence factors, including LasA protease (decreased by 18.8%), pyocyanin (decreased by 63.8%), and LasB elastase (decreased by 42.2%), along with inhibition of biofilm formation and bacterial motility. Molecular docking revealed that its phosphoramidate group interacts with Tyr47 in the LasR L3 loop, disrupting dimer stability and promoting DNA dissociation. Phosphate esterification introduced strong polar interactions, disrupting LasR signaling and quorum sensing. This highlights that polar substituents at C-7 can convert chrysin into an effective antivirulence agent. Ramesh et al. synthesized a series of C7-modified chrysin analogs through epoxide formation at the 7-OH position, followed by ring-opening with various secondary amines [78]. The resulting compounds were evaluated for in vitro antimicrobial activity against Gram-positive and Gram-negative bacteria, as well as fungi. Among them, **53** exhibited the most potent antibacterial and antifungal activity, with zones of inhibition >20 mm and MIC values of 4.68–9.37 μg/mL, while several others showed moderate effects. Molecular docking studies against *E. coli* FabH (PDB ID: 1HNJ) and *Saccharomyces cerevisiae* (*S. cerevisiae*) (PDB ID: 5EQB) supported the experimental findings, with binding scores correlating to the observed antimicrobial activity. Epoxide opening with bulky secondary amines produced derivatives with an enhanced spectrum of activities. Structural flexibility at the 7-position enabled stronger FabH and fungal enzyme inhibition. Kumari et al. synthesized a series of 7-O-piperazine-substituted chrysin analogs and evaluated their antibacterial activities against two Gram-positive strains, *Streptococcus pyogenes* (*S. pyogenes*) and *Staphylococcus aureus* (*S. aureus*)*,* as well as two Gram-negative strains, *E. coli* and *P. aeruginosa* [79]. Among them, compound **54a** showed strong activity against *S. pyogenes* (MIC 12.5 µg/mL), while **54b** was highly active against *E. coli* (MIC 12.5 µg/mL). In contrast, **54c** exhibited moderate antibacterial activity (MIC 125 µg/mL) but showed the highest docking affinities toward *S. aureus* TyrRS (−6.806 kcal/mol) and *E. coli* DNA GyrB (−7.129 kcal/mol). Piperazine substitution significantly enhanced antibacterial potency, though activity varied by bacterial strain. Docking results suggest that affinity for TyrRS and GyrB does not always directly translate to MIC potency, indicating complex SAR. Omonga et al. developed a novel synthetic method, including microwave-assisted O-alkylation, to prepare a series of chrysin derivatives [80]. Among them, the dinitrophenyl-substituted analog **55** exhibited the most potent antibacterial activity, with MIC values ranging from 25 to 62.5 µg/mL against multiple strains, including methicillin-resistant *S. aureus*, *P. aeruginosa*, *Klebsiella pneumoniae* (*K. pneumoniae)*, *E. coli*, and *E. faecalis*. Compared with chrysin, compound **55** demonstrated up to a 20-fold increase in activity, demonstrating the impact of nitro substitution on antibacterial potency. Introduction of a strongly electron-withdrawing dinitrophenyl group markedly boosted antibacterial potency, particularly against resistant strains. This suggests that nitroaromatic substitution can serve as a key driver of activity enhancement.

### 3.4. Antioxidant Activity

The Gençkal group reported heteroleptic Cu(II) complexes incorporating 2,2′-bipyridine or substituted 1,10-phenanthrolines [81]. 2,2′-azino-bis(3-ethylbenzothiazoline-6-sulfonic acid) (ABTS) assays revealed that all Cu(II)–chrysin complexes exhibited significantly higher antioxidant capacities than free chrysin. The bathophenanthroline-containing complex **56** showed the highest activity, likely due to enhanced hydrogen atom transfer efficiency after complexation. Metal coordination with electron-rich ligands enhanced radical scavenging capacity. In particular, bulky phenanthroline derivatives improved hydrogen atom transfer, correlating ligand electronics with superior antioxidant effects. Yang et al. utilized the intrinsic antioxidant property of chrysin to develop multifunctional anti-Alzheimer’s agents [82]. Several chloroethylamines were reacted with the 7-hydroxyl group of chrysin to form ether linkages, and the products were chelated with CuCl_2_ to yield mononuclear complexes. The derivatives and their Cu(II) complexes exhibited strong antioxidant activity and selective butyrylcholinesterase inhibition with submicromolar IC_50_ values for both Acetylcholinesterase (AChE) and Butyrylcholinesterase (BuChE). They also effectively inhibited Aβ_1–42_ aggregation, particularly in the Cu^2+^-induced mode. With low cytotoxicity and favorable in silico predictions for BBB penetration and oral bioavailability, compound **57** was identified as a promising lead for further anti-AD drug development. Introduction of ether linkers and Cu(II) chelation enhanced both antioxidant and anti-amyloid properties. Mononuclear complexes outperformed monomers, indicating that multivalent binding motifs are advantageous for multifunctional AD leads. Liu et al. synthesized novel chrysin carbamate derivatives by reacting the 5- and 7-hydroxyl groups of chrysin with four different carbamyl chlorides [83]. The piperidine-containing analog **58** showed potent butyrylcholinesterase inhibition (IC_50_ = 0.0353 ± 0.0011 µM), with over 1000-fold selectivity over acetylcholinesterase. It also exhibited strong hydroxyl and superoxide radical scavenging activity and was able to release free chrysin via BuChE-mediated hydrolysis, which chelated Cu^2+^ and Fe^2+^ and more effectively inhibited Aβ_1–42_ fibril formation. Given that oxidative stress contributes to neuronal damage and Aβ aggregation in Alzheimer’s disease, the antioxidant property supports its potential as a multifunctional lead for AD therapy. Carbamate substitution at 5,7-positions conferred high BuChE selectivity and radical scavenging ability. The piperidine carbamate further enabled prodrug-like release of chrysin, coupling antioxidant and anti-amyloid effects. Later, the same group reported a protonated chrysin derivative complexed with Cu(II) [84]. Compound **59** exhibited enhanced antioxidant activity along with potent inhibition of Aβ_1–42_ aggregation and cholinesterase. Single-crystal X-ray analysis further provided structural insights, thereby validating its potential as a multi-target anti-AD agent. Protonation improved chelation with Cu(II), stabilizing radical scavenging and Aβ-binding interactions. The crystallographic data highlight how subtle protonation and coordination changes can fine-tune multi-target activity. Recently, the Byun group has utilized hydroxyethylated chrysin **44**, a γ-irradiation-derived chrysin derivative [85]. In antioxidant assays, HE-chrysin showed higher activity than chrysin in 2,2-diphenyl-1-picrylhydrazyl (DPPH), ABTS, and Ferric Reducing Antioxidant Power (FRAP). At 2.5 μM in B16F10 cells, it reduced H_2_O_2_-induced ROS and suppressed IBMX-stimulated melanin production by inhibiting tyrosinase activity and downregulating TRP-1/2 expression, without cytotoxicity. In silico docking revealed stronger binding to tyrosinase (ΔGbind = −7.00 kcal/mol) than chrysin (−6.39) or arbutin (−5.12) through H-bonds with Glu256 and Asn260 and Cu metal chelation. Hydroxyethyl substitution increased both radical scavenging and tyrosinase inhibition. Radiation-derived modification enhanced binding affinity, illustrating that minor hydroxylation changes can markedly elevate antioxidant and antimelanogenic effects.

### 3.5. Anti-Diabetic Activity

Zhang et al. reported the design, synthesis, and mechanistic evaluation of 1-deoxynojirimycin–chrysin conjugates for managing type 2 diabetes [86]. The derivatives were synthesized by first introducing bromoalkyl linkers of varying lengths at the 7-position of chrysin, followed by substitution with 1-deoxynojirimycin. Among the synthesized derivatives, compound **60** exhibited the highest lipophilicity and demonstrated exceptional α-glucosidase inhibitory activity (IC_50_ = 0.51 ± 0.02 µM), approximately 16-fold stronger than 1-deoxynojirimycin. Kinetic and docking studies indicated reversible, mixed-type inhibition with strong binding to the catalytic site through hydrogen bonding and hydrophobic interactions. Linker length and lipophilicity played critical roles, with longer alkyl linkers improving catalytic site interactions. Hybridization with 1-deoxynojirimycin significantly enhanced potency compared with either pharmacophore alone. The Chavasiri group reported the design and synthesis of twenty-three chrysin derivatives via alkylation and bromination reactions [87]. Most derivatives exhibited stronger α-glucosidase inhibitory activity than acarbose. Among them, compounds **61a**, **61b**, and **62** showed remarkable potency with IC_50_ values of 0.08, 3.47, and 2.97 μM, respectively. Enzyme kinetics analysis revealed that 61a functioned as a competitive inhibitor, while **61b** and **62** displayed mixed-type inhibition. The introduction of brominated substituents boosted α-glucosidase inhibition. The comparison of **61a** versus **61b**/**62** illustrates that competitive versus mixed inhibition modes can be tuned by structural variation. The Noh group developed 7-prenylated chrysin derivatives, including chrysin 5-benzyl-7-prenylether (compound **63a**) and chrysin 5,7-diprenylether (compound **63b**), which exhibited improved pharmacological profiles compared with chrysin [88]. Both compounds induced adiponectin secretion in adipogenic human bone marrow mesenchymal stem cells, with compound **63b** showing Peroxisome Proliferator-Activated Receptor gamma (PPARγ) binding affinity comparable to that of pioglitazone and telmisartan. Nuclear receptor binding and ligand-induced coactivator recruitment assays confirmed their role as PPARγ partial agonists. Molecular docking simulations further supported this, revealing interactions within the PPARγ ligand-binding domain consistent with partial agonist activity. Prenylation at the 7-position enhanced hydrophobic interactions and nuclear receptor engagement. The diprenylated analog **63b** showed the strongest PPARγ activity, indicating that multiple prenyl units improve binding affinity and agonist efficacy.

### 3.6. Other Activities

The recent study by the Chavasiri group investigated methoxylated derivatives of chrysin and quercetin for their melanogenesis-stimulating effects [89]. Among more than twenty synthesized flavonoids, 5,7-dimethoxychrysin emerged as the most active chrysin derivative, while 3,3′,4′,5,7-pentamethoxyquercetin was the most potent quercetin derivative, both significantly enhancing melanin production without cytotoxicity. Methoxylation at 5,7 on the A-ring and 3′,4′ on the B-ring was key to promoting melanogenesis, highlighting how substitution patterns modulate pigment-related bioactivity; such methoxy substitution may also improve metabolic stability. A novel chrysin prodrug **65** was synthesized by introducing a hydrophilic group at the 7-hydroxyl position to improve water solubility and prevent rapid phase II metabolism [90]. Compound **65** reduced lipid accumulation, cellular damage, and oxidative stress in non-alcoholic fatty liver disease (NAFLD) model cells, while in db/db mice, it alleviated hyperlipidemia, liver injury, body and liver weight gain, and insulin resistance. Proteomic analysis indicated upregulation of lipid-catabolic and energy-metabolism proteins, and pharmacokinetic studies showed increased oral exposure relative to chrysin. Collectively, these data indicate that hydrophilic 7-O masking improves solubility and reduces rapid phase II conjugation, thereby translating into better pharmacokinetics and efficacy. Falbo et al. explored structural modifications of chrysin to develop novel vascular CaV1.2 channel blockers [91]. Four derivatives, **66a–d**, were synthesized by alkylation and electrophilic aromatic substitution and evaluated using functional assays, electrophysiology, and molecular docking. Methylation of the 5- and 7-hydroxyl groups markedly reduced Ca^2+^ antagonistic potency, while C-8 nitro and amino derivatives retained similar biophysical properties to chrysin and, like nicardipine, bound with high affinity to and stabilized the CaV1.2 channel in its inactivated state. The vasorelaxant effects of these derivatives were vessel-specific, suggesting potential for tailoring chrysin-based scaffolds toward different vascular targets. Free hydroxyl groups at positions 5 and 7 were crucial for CaV1.2 channel modulation. Nitro and amino substitution at C-8 maintained potency, showing that electronic modification can adjust vascular selectivity without loss of core activity.

### 3.7. Promising Chrysin Derivatives Across Therapeutic Domains

A subset of chrysin derivatives can be distinguished as particularly promising scaffolds when evaluated against three rigorous criteria: (i) potency markedly exceeding that of the parent compound, (ii) mechanistic plausibility supported by structure–activity relationships, and (iii) demonstration of efficacy in relevant in vivo models.

In the anticancer domain, the benzimidazole pharmacophore **23** exhibited nanomolar PARP1 inhibition, with binding interactions corroborated by π–π stacking and hydrogen bond analyses. Its therapeutic potential was further established through significant tumor growth suppression in breast cancer xenografts. The spirooxindole carbamate **27** also met these criteria. It showed sub-micromolar cytotoxicity, selective inhibition of CD133^+^ cancer stem-like cells, and induction of apoptosis. The rigid heteroaryl–carbamate framework provided a clear mechanistic rationale, and in vivo studies in HepG2 xenografts confirmed its antitumor activity. In the anti-inflammatory domain, the sulfonyl derivative **45** suppressed NF-κB and STAT3 signaling more effectively than chrysin. The electron-withdrawing sulfonyl group accounted for this effect, and psoriasis models verified its in vivo efficacy. In addition, the α-lipoic acid conjugate **46** combined the flavone core with a redox-active moiety, which enhanced both antioxidant and anti-inflammatory properties. A TNBS-induced colitis rat model confirmed its therapeutic relevance. The hydroxyethyl-substituted derivative **44** can form either through radiation of chrysin or through conventional synthesis (Scheme 5 and Scheme 6). It induced tolerogenic properties in LPS-stimulated dendritic cells, demonstrating its potential as an immunomodulatory scaffold. Moreover, compound **48** upregulated Toll-interacting protein expression in LPS-stimulated macrophages and protected mice from endotoxin-induced lethal shock. These results also highlighted its in vivo relevance as an anti-inflammatory agent. In the metabolic domain, 7-OH acylated derivative **65** exemplifies the utility of prodrug strategies. It improved aqueous solubility and metabolic stability, which addressed a key limitation of chrysin. In vivo studies in db/db mice demonstrated attenuation of hepatic steatosis and significant improvement in NAFLD-related endpoints, thereby validating this scaffold for metabolic disorders. Based on these criteria, compounds **23, 27**, **44**, **45**, **46**, **48**, and **65** can be regarded as priority scaffolds with strong potential for preclinical development. Their selection reflects an integrated assessment of potency, mechanistic foundation, and translational evidence; they are marked with † in Table 1 and Table 2 for clarity.

## 4. Research Progress on the Synthesis of Chrysin Derivatives

The diverse pharmacological activities of chrysin derivatives are closely linked to the breadth of synthetic methodologies developed over the past few decades. In particular, the ability to modify hydroxyl groups and expand the flavone core has enabled the generation of novel derivatives with improved potency and selectivity. Traditional modifications of chrysin have predominantly targeted O-functionalization at the 5- and 7-hydroxyl groups, but this strategy inherently restricts structural diversity. To overcome these limitations and expand the accessible chemical space, alternative synthetic methodologies have been developed that allow direct functionalization at carbon positions of the flavone backbone. These approaches have facilitated the preparation of structurally novel derivatives with potentially enhanced or diversified biological activities. In this section, we highlight such methodologies, with particular emphasis on C–C bond-forming strategies, utilizing transition-metal-catalyzed coupling reactions.

### C-C Bond Formation via Transition-Metal-Mediated Coupling Reactions

**Scheme 8:**
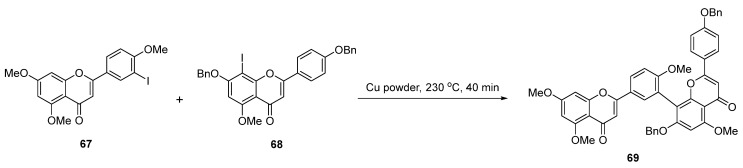
Synthesis of biflavonoid via the Ullmann reaction.

One of the most widely used strategies for constructing new flavonoid derivatives via direct carbon–carbon (C–C) bond formation is transition-metal-catalyzed cross-coupling reactions, including Ullmann [92], Suzuki–Miyaura [93,94], Stille [95], and Sonogashira reactions [96]. Ullmann reaction, catalyzed by copper, enables the coupling of aryl iodides and has been effectively employed for the dimerization of iodinated flavonoid intermediates. A representative example is the synthesis of the biflavonoid ginkgetin (Scheme 8). In this process, compounds **67** and **68** undergo coupling under high-temperature conditions with copper powder to afford **69**, and subsequent debenzylation yields ginkgetin [97,98]. The Ullmann reaction offers a simple and efficient route for generating bis-flavonoid scaffolds; however, it generally requires elevated temperatures and activated copper, often leading to undesired side products that complicate purification and reduce overall yield.

**Scheme 9:**
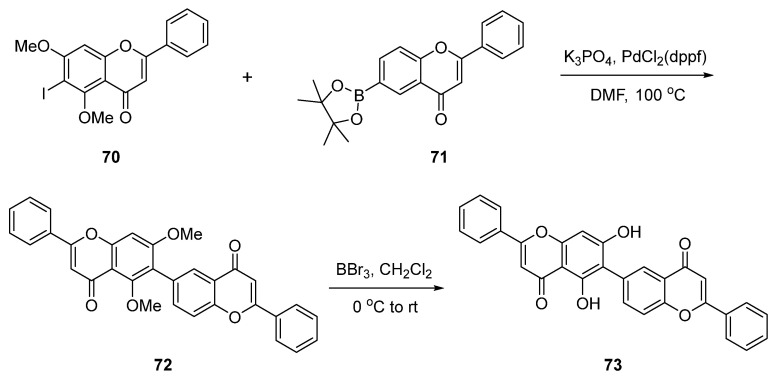
Synthesis of biflavone **73** via the Suzuki–Miyaura coupling reaction.

The Suzuki–Miyaura reaction, a palladium-catalyzed cross-coupling between aryl halides and aryl boronates, is widely recognized for its robustness, selectivity, and broad functional group tolerance under mild conditions. For application to chrysin derivatives, halogenated and boronate-substituted intermediates must first be prepared (Scheme 9), which can then undergo palladium-catalyzed coupling to form new C–C bonds in a chemoselective and regioselective manner. This strategy substantially expands the accessible chemical space of chrysin derivatives. Kim et al. employed this methodology to synthesize biflavone **72** from the 6-iodinated chrysin intermediate **70** and the boronate-substituted flavone **71** (Scheme 9) [99]. Next, deprotection of *O*-methyl groups using BBr_3_ provided the final product **73**. This compound exhibited pronounced anti-inflammatory activity, strongly inhibiting COX-2-mediated PGE_2_ production (IC_50_ = 0.11 μM, ~38-fold more potent than the parent structure) without altering COX-2 expression. It also reduced NO production (46.9% at 50 μM) and demonstrated significant in vivo efficacy, attenuating carrageenan-induced paw edema and acetic acid-induced pain in mice.

**Scheme 10:**
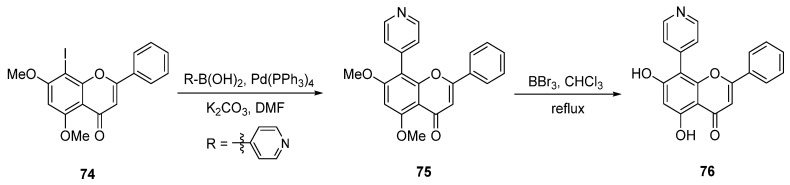
Synthesis of 5,7-dihydroxy-8-(pyridine-4-yl) chrysin via the Suzuki–Miyaura coupling reaction.

Subsequently, the same group extended this strategy to the synthesis of 8-aryl-substituted chrysin derivatives by coupling the 8-iodoflavone intermediate **74** with various boronic acids under Pd(PPh_3_)_4_/K_2_CO_3_ conditions (Scheme 10) [100]. Among the synthesized products, 5,7-dihydroxy-8-(pyridin-4-yl)chrysin **76** displayed markedly improved activity over chrysin, significantly inhibiting COX-2-mediated PGE_2_ production and inducible nitric oxide synthase (iNOS)-dependent NO release in LPS-stimulated RAW 264.7 cells at 10 μM. Detailed evaluation revealed IC_50_ values of 6.2 μM (PGE_2_) and 22.6 μM (NO), together with potent suppression of the pro-inflammatory cytokines TNF-α and IL-6 (10–50 μM) [101]. In vivo, compound **76** reduced λ-carrageenan-induced paw edema (25.2–44.3% inhibition at 10–100 mg/kg) and attenuated collagen-induced arthritis at 50 mg/kg, suggesting its potential as a novel anti-inflammatory candidate.

**Scheme 11:**
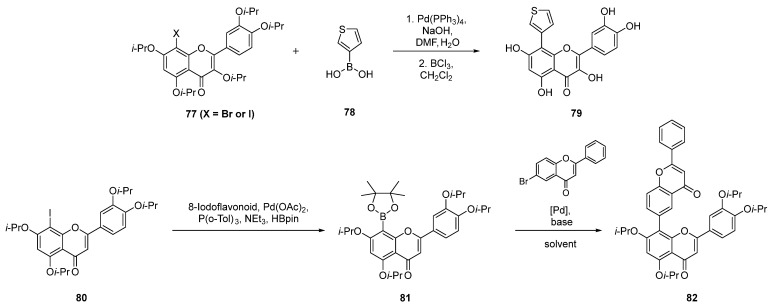
Synthesis of the synthetic flavonol **82** via the Suzuki–Miyaura coupling reaction.

In 2022, Křen et al. reported the synthesis of diverse unnatural flavonoid derivatives via the Suzuki–Miyaura cross-coupling reaction [102]. Specifically, halogenated flavonol intermediate **77** was coupled with aryl boronic acid **78** to generate structurally novel products (Scheme 11). The authors also optimized borylation conditions for halogenated flavonols, affording the borylated intermediate **81**, which was subsequently employed in cross-coupling with brominated flavones to access a broad array of unnatural flavone scaffolds (Scheme 11).

**Scheme 12:**
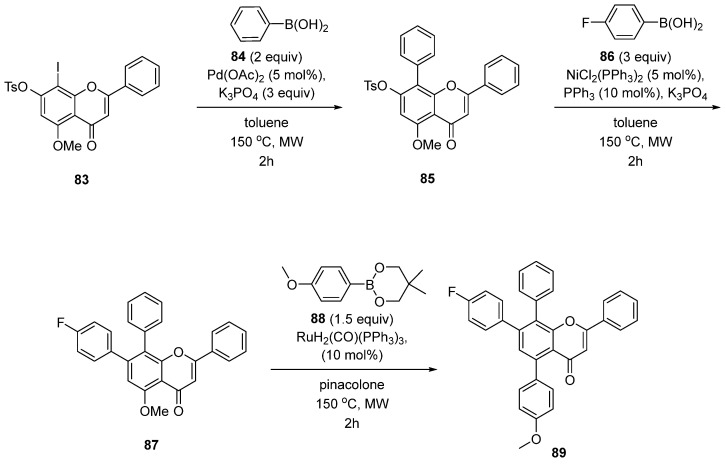
Chemoselective polyarylation of flavone **89** via sequential transition-metal-mediated coupling reactions.

Recently, da Silva et al. reported a chemoselective strategy for synthesizing aryl-substituted flavones from chrysin using transition-metal-catalyzed reactions, enabling aryl substitutions at positions 5, 5,8, 5,6, and 5,6,7 [103]. A key feature of this approach is the site-selective functionalization of the flavone backbone, dictated by the choice of catalyst and reaction conditions. As shown in Scheme 12, chrysin was first modified by tosylation and methylation at the 7- and 5-hydroxy groups, followed by iodination at C-9 to give the intermediate **83**. Subsequent cross-coupling reactions catalyzed by palladium, nickel, or ruthenium selectively introduced aryl groups at the 9-, 7-, or 6-positions, respectively. Compound **89** was obtained as the final product through a sequence of three successive transition-metal-catalyzed cross-coupling reactions. Owing to the ready availability of chrysin and the operational simplicity of these transformations, this methodology expands the synthetic accessibility of structurally diverse chrysin derivatives. Notably, however, the biological activities of these new derivatives were not reported.

**Scheme 13:**
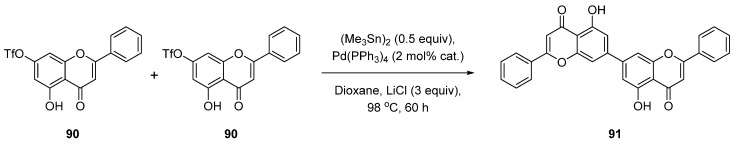
Synthesis of 7,7″-bis-chrysin **91** via the Stille coupling reaction.

Stille coupling, another palladium-catalyzed cross-coupling reaction, has been less frequently applied to flavonoid synthesis than the Suzuki–Miyaura methodology. This transformation typically couples flavonoid triflates **90** with organostannane reagents in the presence of a palladium catalyst and base. Using distannane reagents, symmetrical bis-flavone derivatives can be obtained, as exemplified by the synthesis of the unnatural 7,7″-bis-chrysin dimer 91 (Scheme 13) [104].

**Scheme 14:**
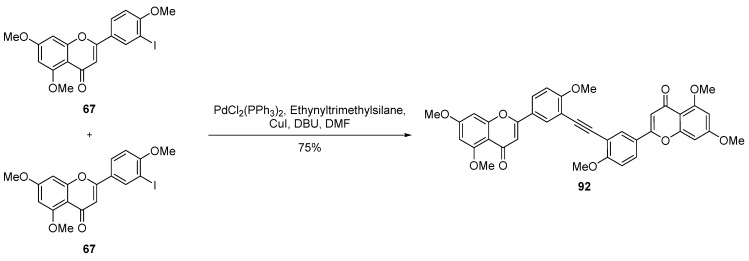
Synthesis of bi-isoflavone **92** via the Sonogashira coupling reaction.

Sonogashira coupling, which employs palladium and copper co-catalysts, has also been applied to the synthesis of novel flavone derivatives from iodinated flavones. Deodhar et al. reported the preparation of a range of bi-isoflavones and bisflavones through this strategy (Scheme 14) [97]. Iodinated compound **67** reacted with ethynyltrimethylsilane in the presence of palladium and copper catalysts to afford product **92**. Unlike other cross-coupling methods, the Sonogashira reaction does not generate direct C–C linkages within the flavone backbone; rather, it introduces an alkyne moiety as a linker between flavone units, thereby affording structurally distinct flavonoid architectures.

**Scheme 15:**
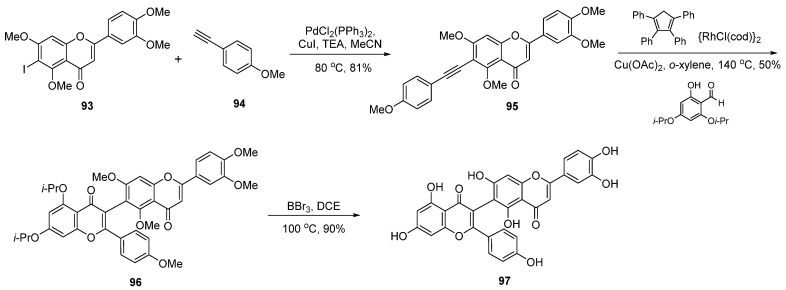
Synthesis of ridiculuflavone **97** via the Sonogashira coupling reaction.

Ridiculuflavone A and I3,II8-bis-apigenin were synthesized using the Sonogashira coupling strategy [105]. In this study, direct Suzuki–Miyaura coupling of two flavone units was unsuccessful, but coupling of an iodinated flavone derivative **93** with a phenylacetylene **94** via the Sonogashira reaction yielded an alkyne-containing intermediate **95** (Scheme 15). Subsequent oxidative cyclization catalyzed by rhodium in the presence of 2-hydroxy-4,6-diisopropoxybenzaldehyde and 1,2,3,4-tetraphenyl-1,3-cyclopentadiene yielded a new flavone product **96**. Final demethylation with BBr_3_ yielded ridiculuflavone A **97**. This synthetic route is distinct from traditional methods in that it employs Sonogashira coupling to generate an alkyne precursor, followed by oxidative cyclization, rather than forming a C–C bond between two flavone moieties.

## 5. Perspective

Over the past few decades, significant progress has been made in the synthesis and biological evaluation of chrysin derivatives. A wide spectrum of structural modifications has been reported, many of which confer enhanced pharmacological properties compared with the parent compound. These advances clearly highlight the potential of chrysin as a versatile scaffold for drug discovery and functional material development. From a synthetic standpoint, diverse methodologies—including etherification, esterification, sigmatropic rearrangements, and condensation—have been widely employed, while the advent of transition-metal-catalyzed reactions has been particularly transformative. These approaches enable the construction of C–C bonds at positions on the flavone core that are inaccessible through traditional transformations, thereby opening new chemical spaces for chrysin-based analogs. Future studies are expected to further exploit these methodologies, generating unprecedented structural variants that may harbor unique or superior bioactivities.

Despite these advances, current pharmacological evaluations remain largely confined to the cellular level. To ensure translational relevance, comprehensive pharmacological assessments in animal models—including efficacy, chronic toxicity, and disease-specific applications—will be essential. Such studies will help bridge the gap between promising in vitro activity and preclinical development. Equally important is the need to systematically address the pharmacokinetic limitations of chrysin derivatives, such as low solubility, rapid metabolism, and poor oral bioavailability. Innovative formulation approaches—such as nanoparticle encapsulation, liposomal carriers, and conjugation with functional nanostructures—offer promising solutions to enhance solubility, prolong systemic circulation, and enable targeted delivery.

Looking forward, the integration of artificial intelligence (AI) and machine learning (ML) is expected to accelerate the identification and optimization of promising flavonoid derivatives [106,107]. By incorporating synthetic accessibility, SAR patterns, and pharmacological profiling into predictive algorithms, AI can guide rational scaffold design and prioritize derivatives with improved therapeutic indices [108]. At the same time, molecular imaging approaches using radiolabeled derivatives will play a pivotal role in validating translational potential. Incorporation of isotopes such as ^14^C and ^3^H allows direct assessment of absorption, distribution, metabolism, and excretion (ADME) without altering the native structure of chrysin derivatives, making them ideal for quantitative whole-body autoradiography (QWBA) and liquid scintillation studies [109,110,111]. Such tracer studies can provide critical insights into tissue distribution, clearance, and metabolic fate in animal models, thereby establishing the pharmacokinetic foundation required for drug development.

Taken together, the future of chrysin research will depend on three converging strategies First, synthetic creativity should be expanded to access structurally diverse and biologically active derivatives. Second, comprehensive pharmacological validation should be secured in animal models, including imaging-based ADME assessment with ^14^C and ^3^H tracers. Finally, innovative formulation technologies and AI-assisted design need to overcome pharmacokinetic barriers and guide rational optimization.

Through these combined efforts, chrysin derivatives may ultimately evolve from laboratory curiosities into clinically relevant therapeutic agents and multifunctional platforms for disease management and diagnostics. By integrating advances in synthetic chemistry, computational modeling, nanodelivery technologies, and molecular imaging, chrysin is poised to serve not only as a promising natural product scaffold but also as a foundation for next-generation therapeutics with tangible translational impact.

## Figures and Tables

**Table 1:** Structures, synthesis methods, and antitumor activities (IC_50_) of chrysin derivatives.

Entry	Structure	Synthesis Method	Cell Lines (IC_50_)	Key Findings and Mechanisms	**Ref**
**1**	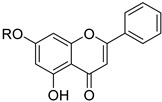 **2a**: R = C_2_H_5_ **2b**: R = C_3_H_7_ **2c**: R = CH(CH_3_)CO_2_H	Etherification (7-OH)	For **2c**: Vero (152.28 ± 3.82 µM), HeLa (13.91 ± 0.34 µM), A549 (147.38 ± 7.56 µM)	7-OH substitution altered enzyme selectivity. Compound **2c** inhibited HDAC8, increased H3 acetylation, and induced apoptosis.	[37]
**2**	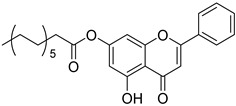 **3**	Esterification (7-OH)	HepG2 (14.79 µM)	Long-chain myristoyl ester improved solubility. Enhanced membrane permeability translated into stronger cytotoxicity in HepG2 cells.	[23]
**3**	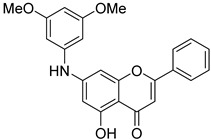 **4**	Etherification with *N*-(chloroacetyl) aniline analogs (7-OH) followed by Smiles rearrangement	GI_50_ values: MCF-7 (0.03 µM), HCT-15 (0.06 µM)	Smiles rearrangement gave a diphenylamine scaffold. Stronger π–π interactions yielded nanomolar potency in breast and colon cancer cells.	[22]
**4**	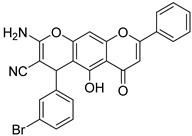 **6**	Michael addition followed by cyclization (Scheme 1)	K562 (6.41 ± 0.49 µM)	Chromene–chrysin hybrid induced mitochondrial apoptosis. Regulated Bax/Bcl-2 balance and suppressed tumor growth in vivo.	[38]
**5**	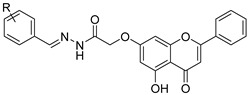 **7a**: R = 2,4-OH **7b**: R = 2,3,4-OH	Etherification (7-OH), hydrazide formation, and hydrazone condensation	For **7a**: MDA-MB-231 (5.98 µM) For **7b**: MDA-MB-231 (9.40 µM)	PAC-1 hybrids induced G2 arrest and apoptosis. Triggered Bak upregulation, cytochrome c release, and caspase activation.	[39]
**6**	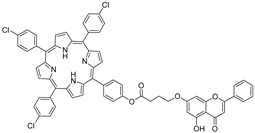 **8**	Etherification (7-OH) followed by coupling with porphyrin analogs	HeLa (6.26 ± 2.52 µM), A549 (23.37 ± 4.24 µM)	Porphyrin–chrysin hybrid acted as a photosensitizer. Positive charge and EWG groups enhanced phototoxicity under irradiation.	[40]
**7**	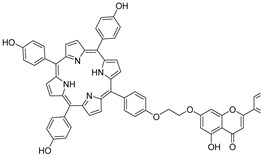 **9**	Etherification (7-OH) followed by conjugation with porphyrin derivatives	MGC-803 (70.41 ± 2.15 µM), HeLa (26.51 ± 1.15 µM)	Porphyrin conjugates bound ct-DNA and generated ROS. Free-base derivatives outperformed metalated analogs in terms of photodynamic efficiency.	[41]
**8**	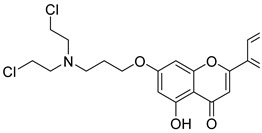 **10**	Etherification (7-OH), substitution with diethanolamine, and chlorination	HeLa (1.43 µM), A549 (7.34 µM), HepG2 (11.31 µM), MCF-7 (4.90 µM), SH-SY5Y (7.86 µM), PC-3 (2.32 µM), DU145 (2.91 µM)	Nitrogen mustard–chrysin hybrids disrupted ΔΨm. Three-carbon linker produced the strongest cytotoxic effect.	[42]
**9**	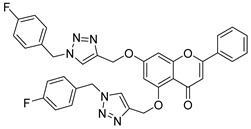 **12**	Propargylation (5,7-OH) followed by click reaction	HeLa (0.7331 µM), SiHa (1.352 µM)	Bis-triazole substitution improved H-bonding. Compound **12** achieved submicromolar IC_50_ in HeLa cells.	[43]
**10**	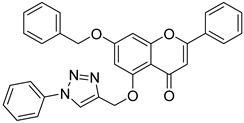 **13**	Propargylation (5-OH) followed by click reaction	PC3 (10.8 ± 0.04 µM), MCF-7 (20.5 ± 0.2 µM)	Phenyl-triazole derivative enhanced π–π stacking. Showed potent activity in PC3 and MCF-7 cells.	[44]
**11**	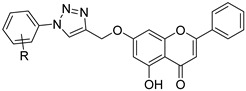 **14a**: R = 3-F **14b**: R = 2-Cl	Propargylation (7-OH) followed by click reaction	For **14a**: MGC-803 (18.40 µM) For **14b**: MGC-803 (5.92 µM)	Halogen-substituted triazoles improved activity. Chloro- and fluoroaryl groups boosted gastric cancer cell potency.	[45]
**12**	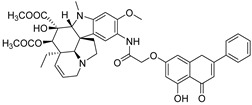 **15**	Conjugation with a vindoline derivative (7-OH) followed by Smiles rearrangement	GI_50_ values: low micromolar levels for 60 human cancer cells	Vindoline–chrysin hybrids combined dual pharmacophores. Flexible linker design gave broad low-micromolar GI_50_ across the NCI-60 panel.	[46]
**13**	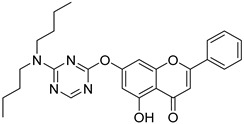 **18**	Nucleophilic aromatic substitution with 1,3,5-triazine derivatives (7-OH) (Scheme 3)	HeLa (9.86 ± 0.37 µM), 293 T (37.34 ± 1.28 µM)	Triazine–chrysin hybrids introduced electron-deficient heteroaryl moieties. Compound **18** outperformed cisplatin in HeLa cells.	[47]
**14**	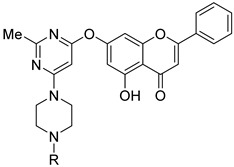 **19a**: (R =1-methyl piperazine) **19b**: (R = methyl)	Nucleophilic aromatic substitution with 4,6-dichloropyrimidine derivatives (7-OH)	For **19a**: A549 (30.30 µM), HepG2 (21.02 µM), MCF-7 (24.67 µM), PC-3 (22.130 µM) For **19b**: HCT-116 (4.83 µM)	Pyrimidine–chrysin hybrids showed strong cytotoxicity. Compound **19b** was especially potent against HCT-116 cells.	[48]
**15**	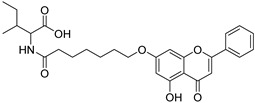 **20**	Etherification (7-OH) followed by coupling with L-amino acids	MGC-803 (24.5 ± 3.4 µM),	Heptanoyl–L-amino acid conjugate increased solubility. Compound **20** showed improved efficacy in gastric cancer.	[49]
**16**	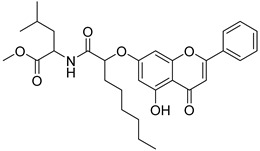 **21**	Etherification (7-OH) followed by coupling with L-amino esters	MCF-7 (32.4 ± 1.8 µM), MDA-MB-231 (8.2 ± 1.6 µM)	Leucine methyl ester conjugate induced G2/M arrest. Activated mitochondrial apoptosis and inhibited Akt phosphorylation.	[50]
**17**	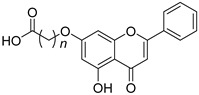 **22a**: *n* = 4 **22b**: *n* = 5 **22c**: *n* = 6	Etherification (7-OH)	Vero (39.94~49.55 µM), HeLa (15.96~18.00 µM), HCT-116 (11.96~13.04 µM), A549 (40.0~41.48 µM), MCF-7 (21.82~37.28 µM)	Carboxylated derivatives targeted the HDAC active site. Zn^2+^ chelation supported inhibitory mechanism.	[51]
**18 †**	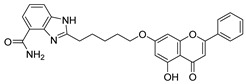 **23**	Etherification (7-OH) followed by coupling with a benzimidazole derivative	MDA-MB-231 (6.2 ± 0.6 µM), MDA-MB-436 (4.5 ± 1.4 µM), HCC-1937 (3.9 ± 0.8 µM), MCF-7 (14.2 ± 1.1µM)	Benzimidazole pharmacophore enhanced PARP1 binding. Compound **23** inhibited BRCA-deficient breast cancer cells at nanomolar levels.	[52]
**19**	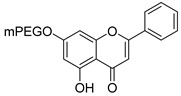 **24a**: (mPEG 350 g/mol) **24b**: (mPEG 500 g/mol) **24c**: (mPEG 750 g/mol) **24d**: (mPEG 2 kDa)	Etherification (7-OH)	PC-3 (205.66 ± 2.60 µM), HepG2 (122.72 ± 9.92 µM)	PEGylation improved solubility and aggregation. mPEG-500 conjugate retained cytotoxic and redox activity.	[53]
**20**	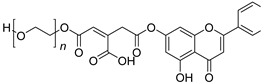 **25**	Esterification (7-OH) with cis-aconityl functionalized PEG_4000_	MDA-MB-231 (6.2 ± 2.3 µM), MCF-7 (26.1 ± 3.5 µM)	PEG4000–cis-aconityl conjugate enabled pH-sensitive release. Showed enhanced efficacy in breast cancer cells.	[54]
**21 †**	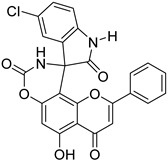 **27**	Formation of spirooxindole carbamate derivatives (Scheme 4)	A549 (24.20 ± 2.60 µM), MDA MB-231 (24.93 ± 2.50 µM), HepG2 (2.50 ± 0.25 µM), HeLa (7.48 ± 1.23 µM), HEK-293 (14.25 ± 3.60 µM)	Spirooxindole carbamate bound to CD133 protein. Reduced CSC populations and inhibited tumorsphere formation.	[59]
**22**	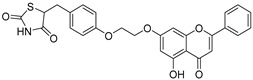 **28**	Etherification (7-OH) followed by conjugation with a thiazolidine-2, 4-dione derivative	Anticancer agent as a TLR4 ligand	Thiazole derivative acted as a TLR4 ligand. Activated NF-κB signaling and reprogrammed TAMs to the M1 phenotype.	[60]
**23**	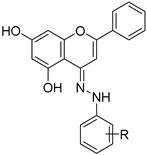 **29a**: R = 2-OMe **29b**: R = 4-OMe	Hydrazone formation (condensation)	for **29a**: MCF-7 (179.28 µM), HepG2 (166.19 µM) for **29b**: MCF-7 (101.44 µM), HepG2 (236.49 µM)	Hydrazone derivatives showed moderate cytotoxicity. Activity varied with methoxy substitution on phenylhydrazine.	[61]
**24**	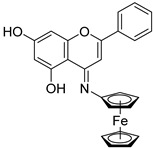 **30**	Imine formation (condensation)	HepG2 (3.11 ± 0.41 µM), HCT-116 (3.47 ± 0.69 µM), A549 (3.35 ± 0.27 µM), MCF-7 (14.08 ± 0.96 µM)	Ferrocene Schiff base inhibited Topo II. Induced ROS-mediated apoptosis with ~5-fold selectivity over normal cells.	[64]
**25**	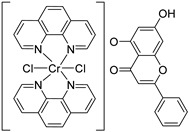 **31**	Chelation with Cr(III) mixed-ligand complexes	MCF-7 (8.08 µM)	Cr(III)–phenanthroline–chrysin complex increased uptake. Showed higher cytotoxicity than free chrysin.	[65]
**26**	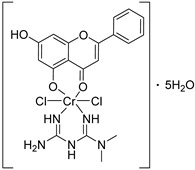 **32**	Chelation with Cr(III) mixed-ligand complexes	MCF-7 (30.85 µM)	Cr(III)–metformin–chrysin complex altered polarity. Demonstrated improved activity but less than that of compound **31**.	[65]
**27**	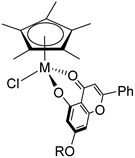 **33a**: (M = Ru), **33b**: (M = Rh) **33c**: (M = Ir) R = −(CH_2_)_4_-piperidine	Chelation with transition metal complexes	For **33a**: SW480 (28.5 ± 1.3 µM), A549 (31.3 ± 1.6 µM) For **33b**: SW480 (31.3 ± 1.3 µM), A549 (35.3 ± 1.0 µM) For **33c**: SW480 (15.9 ± 1.3 µM), A549 (18.9 ± 1.1 µM)	Half-sandwich Ru, Rh, Ir complexes showed distinct profiles. Ir(III) complex induced ROS and mitochondrial apoptosis with high selectivity.	[66]
**28**	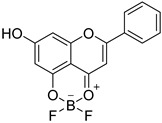 **34**	Chelation with boron difluoride	MCF-7 (29.7 µM), SKOV3 (31.9 µM)	Difluoroboron complex modified electronic distribution. Showed >2-fold improved cytotoxicity versus parent chrysin.	[67]

† Prioritized scaffold, selected based on the following criteria: (i) potency superior to parent chrysin, (ii) mechanistic rationale supported by SAR, and (iii) efficacy demonstrated in in vivo models.

**Table 2:** Structures, synthesis methods, biological activities, and key mechanisms of chrysin derivatives.

Entry	Structure	Synthesis Methods	Biological Activity	Key Findings and Mechanisms	**Ref**
**1**	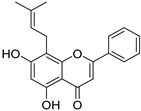 **40**	Claisen rearrangement of prenyl group (5-OH)	Anti-inflammatory	Inhibited COX-2 (IC_50_ = 6.76, 9.63 μM vs. chrysin, 18.48 μM) in RAW264.7 cells, reducing IL-6, TNF-α, and PGE_2_. Docking showed strong COX-2 binding via H-bonds/hydrophobic contacts, confirming selective inhibition in vitro.	[68]
**2**	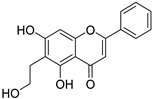 **42**	Claisen rearrangement of allyl group (5-OH)	Anti-inflammatory		[68]
**3** ** †**	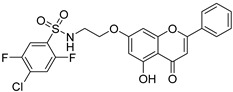 **45**	Etherification (7-OH) followed by sulfonylation	Anti-inflammatory/ Anti-psoriasis	Suppressed NO, TNF-α, IL-6, and IL-17A (IC_50_ = 8.0 μM vs. dexamethasone, 19.5 μM) in RAW264.7 and HaCaT cells. Reduced epidermal thickness and cytokine levels in mice with imiquimod-induced psoriasis through NF-κB/STAT3 inhibition.	[69]
**4** ** †**	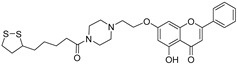 **46**	Etherification (7-OH) followed by conjugation with α-lipoic acid	Anti-inflammatory	Inhibited monocyte adhesion to colon epithelial cells (IC_50_ = 4.71 μM vs. α-LA, 12.7 μM). Ameliorated TNBS-induced colitis in rats by suppressing ICAM-1/MCP-1 expression and JAK2/STAT3 signaling.	[70]
**5**	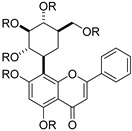 **47a**: R = Ac **47b**: R = CO_2_Et	**47a**: Acetylation using acetic anhydride **47b**: Carbonylation using ethyl chloroformate	Anti-inflammatory/ Antioxidant	Reduced ROS, TNF-α, IL-1β, and COX-2 in LPS-stimulated THP-1 macrophages. Activated Keap1/Nrf2/HO-1; 47a showed the strongest effect, supporting antioxidant/anti-inflammatory dual action.	[71]
**6** ** †**	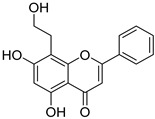 **44**	γ-radiation	Anti-inflammatory	Reduced cytokine release (TNF-α, IL-6, NO) in RAW264.7 macrophages by upregulating Tollip. In vivo, alleviated dermatitis and endotoxin-induced shock in mice by suppressing NF-κB/MAPK pathways.	[72,73]
**7** ** †**	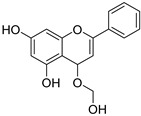 **48**	γ-radiation	Anti-inflammatory		[74]
**8**	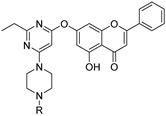 **49a**: 2,3-dichlorophenyl **49b**: diphenyl methyl	Nucleophilic aromatic substitution with 4,6-dichloropyrimidine derivatives (7-OH) followed by substitution with piperazine analogs	Antimicrobial	Displayed strong antibacterial activity against *E. coli* (MIC 6.25–12.5 μg/mL) and antifungal effects against *C. albicans*. Docking confirmed binding to *E. coli* DNA gyrase and *C. albicans* CYP51, outperforming standard antibiotics.	[75]
**9**	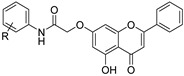 **50a**: R = 3-Cl **50b**: R = 4-F **50c**: R = 2-OCH_3_	Conjugation with *N*-(chloroacetyl) aniline analogs (7-OH)	Antimicrobial	Inhibited >92% biofilm formation in *E. coli* MTCC40 at sub-MIC levels (33.57% vs. chrysin). Suppressed motility and improved solubility/bioavailability, enhancing anti-biofilm efficacy.	[76]
**10**	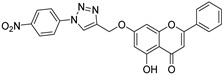 **51**	Propargylation (7-OH) followed by click reaction	Antimicrobial		[76]
**11**	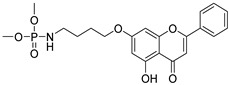 **52**	Etherification (7-OH)	Antimicrobial	Inhibited LasR-regulated virulence factors (LasA, pyocyanin, elastase) and biofilm in *P. aeruginosa*. Docking showed phosphate group binding to Tyr47 in the LasR loop, destabilizing dimerization and DNA binding.	[77]
**12**	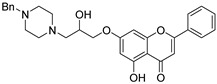 **53**	Etherification (7-OH) with epichlorohydrin, followed by epoxide opening	Antimicrobial	Exhibited broad antibacterial/antifungal activity (MIC 4.68–9.37 μg/mL; inhibition zones > 20 mm). Docking supported binding to *E. coli* FabH and *S. cerevisiae* target enzymes, aligning with experimental potency.	[78]
**13**	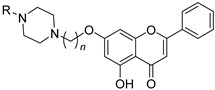 **54a**: *n* = 3, R = 4-methoxyphenyl **54b**: *n* = 3, R = benzyl **54c**: *n* = 4, R = chlorodiphenylmethyl	Etherification (7-OH) followed by nucleophilic substitution with piperazine analogs	Antimicrobial	Active against *S. pyogenes* and *E. coli* (MIC 12.5 μg/mL); weaker activity for **54c**. Docking indicated strong affinity of **54c** for *S. aureus* TyrRS and *E. coli* DNA GyrB despite higher MIC values.	[79]
**14**	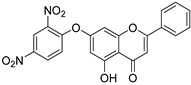 **55**	Etherification (7-OH) under microwave irradiation	Antimicrobial	Showed potent antibacterial activity (MIC 25–62.5 μg/mL) against MRSA, *P. aeruginosa*, *K. pneumoniae*, and *E. coli*. Nitro substitution enhanced potency up to 20-fold versus chrysin, effective even on resistant strains.	[80]
**15**	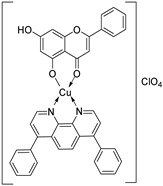 **56**	Chelation with Cu(II)–1,10-phenanthroline complexes	Antioxidant	Demonstrated highest antioxidant capacity in ABTS assays among Cu–chrysin complexes. Improved H-atom transfer efficiency correlated with bulky phenanthroline ligand coordination.	[81]
**16**	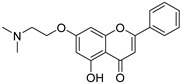 **57**	Etherification (7-OH)	Antioxidant (Anti-Alzheimer)	Showed strong antioxidant and BuChE inhibition (sub-μM IC_50_) in vitro. Reduced Aβ_1–42_ aggregation and predicted BBB penetration, supporting AD therapeutic potential.	[82]
**17**	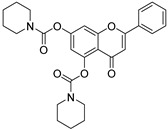 **58**	Acylation with carbamoyl chloride derivatives (5,7-OH)	Antioxidant (Anti-Alzheimer)	Potent BuChE inhibitor (IC_50_ = 0.035 μM, >1000-fold more potent than AChE) with radical scavenging activity. Released free chrysin in vitro, chelating Cu^2+^/Fe^2+^ and blocking Aβ fibril formation.	[83]
**18**	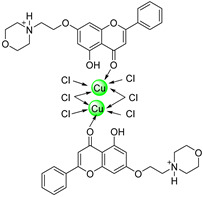 **59**	Etherification with morphine derivative (7-OH) followed by chelation with Cu metal	Antioxidant (Anti-Alzheimer)	Enhanced antioxidant activity and cholinesterase inhibition in vitro. Suppressed Aβ aggregation with structural validation using X-ray crystallography.	[84]
**19**	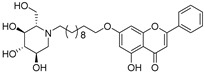 **60**	Etherification (7-OH) followed by conjugation with 1-deoxynojirimycin	Antidiabetic	Strong α-glucosidase inhibition (IC_50_ = 0.51 μ M, 16× DNJ) via mixed-type binding. Docking revealed stable H-bonds and hydrophobic contacts with catalytic site.	[86]
**20**	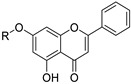 **61a**: R = (CH_2_)_7_CH_3_ **61b**: R = CH_2_CHCH_2_	Etherification (7-OH)	Antidiabetic	Showed superior α-glucosidase inhibition (IC_50_ = 0.08–3.47 μM) vs. acarbose. Compound **61a** was competitive, while **61b** and **62** acted as mixed inhibitors, demonstrating tunable inhibition modes.	[87]
**21**	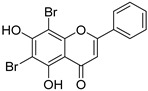 **62**	Bromination	Antidiabetic	α-glucosidase inhibition (IC_50_ = 2.97 ± 0.03 μM) with mixed-type kinetics.	[87]
**22**	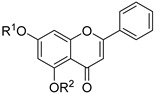 **63a**: R^1^ = prenyl, R^2^ = Bn **63b**: R^1^ = prenyl, R^2^ = prenyl	Etherification (5,7-OH)	Antidiabetic	Stimulated adiponectin secretion in hBMSCs and bound PPARγ. Compound **63b** showed partial agonist activity comparable to that of pioglitazone, validated via docking and coactivator assays.	[88]
**23**	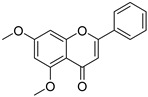 **64**	Etherification (5,7-OH)	Melanogenesis stimulation	Enhanced melanogenesis: 2.7 times increase (1.9 times higher than chrysin).	[89]
**24** ** †**	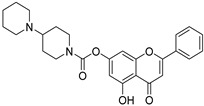 **65**	Acylation with carbamoyl chloride derivatives (7-OH)	Anti-NAFLD	In vitro, it reduced lipid accumulation, oxidative stress, and hepatocellular injury in NAFLD model cells. In vivo, it alleviated hyperlipidemia, liver injury, body and liver weight gain, and insulin resistance in db/db mice.	[90]
**25**	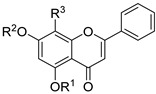 **66a**: R^1^ = H, R^2^ = CH_3_, R^3^ = H **66b**: R^1^ = CH_3_, R^2^ = CH_3_, R^3^ = H **66c**: R^1^ = H, R^2^ = H, R^3^ = NO_2_ **66d**: R^1^ = H, R^2^ = H, R^3^ = NH_2_	**66a**, **b**: Etherification (5,7-OH), **66c**, **d**: Nitration followed by reduction	Vasorelaxant	**66a**: reduced Ca^2+^ antagonism, weak vasorelaxant activity **66b**: minimal Ca^2+^ blocking **66c**: most potent vasorelaxant (pIC_50_ = 5.80, E_max_ ~99%), strong CaV1.2 inhibition **66d**: similar efficacy to chrysin (E_max_ ~93–99%), vessel-selective vasorelaxation.	[91]

† Prioritized scaffold, selected based on the following criteria: (i) potency superior to parent chrysin, (ii) mechanistic rationale supported by SAR, and (iii) efficacy demonstrated in in vivo models.

## Abbreviations

GI_50_	50% growth inhibition concentration
HDAC	Histone deacetylase
PARP	Poly(ADP-ribose) polymerase 1
TLR4	Toll-like receptor 4
COX-2	Cyclooxygenase-2
LPS	Lipopolysaccharide
*C. albicans*	*Candida albicans*
*E. coli*	*Escherichia coli*
*P. aeruginosa*	*Pseudomonas aeruginosa*
*S. aureus*	*Staphylococcus aureus*
*S. cerevisiae*	*Saccharomyces cerevisiae*
*S. pyogenes*	*Streptococcus pyogenes*
ABTS	2,2′-Azino-bis(3-ethylbenzothiazoline-6-sulfonic acid)
AChE	Acetylcholinesterase
BuChE	Butyrylcholinesterase
*K. pneumoniae*	*Klebsiella pneumoniae*
DPPH	2,2-Diphenyl-1-picrylhydrazyl
FRAP	Ferric Reducing Antioxidant Power
NAFLD	Non-alcoholic fatty liver disease
PPARγ	Peroxisome Proliferator-Activated Receptor gamma
iNOS	Inducible nitric oxide synthase
